# Label-free SARS-CoV-2 detection and classification using phase imaging with computational specificity

**DOI:** 10.1038/s41377-021-00620-8

**Published:** 2021-09-01

**Authors:** Neha Goswami, Yuchen R. He, Yu-Heng Deng, Chamteut Oh, Nahil Sobh, Enrique Valera, Rashid Bashir, Nahed Ismail, Hyunjoon Kong, Thanh H. Nguyen, Catherine Best-Popescu, Gabriel Popescu

**Affiliations:** 1 Department of Bioengineering, University of Illinois Urbana-Champaign, Urbana, Illinois 61801 USA; 2 Beckman Institute of Advanced Science and Technology, University of Illinois Urbana-Champaign, Urbana, Illinois 61801 USA; 3 Department of Electrical and Computer Engineering, University of Illinois Urbana-Champaign, Urbana, Illinois 61801 USA; 4 Department of Chemical and Biomolecular Engineering, University of Illinois at Urbana-Champaign, Urbana, IL 61801 USA; 5 Department of Civil and Environmental Engineering, University of Illinois at Urbana-Champaign, Urbana, IL 61801 USA; 6 NCSA Center for Artificial Intelligence Innovation, University of Illinois at Urbana-Champaign, Urbana, IL 61801 USA; 7 Holonyak Micro and Nanotechnology Laboratory, University of Illinois at Urbana-Champaign, Urbana, Illinois 61801 USA; 8 Biomedical Research Center, Carle Foundation Hospital, 509W University Ave., Urbana, Illinois 61801 USA; 9 Carle Illinois College of Medicine, 807 South Wright St., Urbana, Illinois 61801 USA; 10 Mayo-Illinois Alliance for Technology Based Healthcare, Urbana, Illinois 61801 USA; 11 Department of Pathology, College of Medicine, University of Illinois at Chicago, Chicago, IL USA

**Keywords:** Interference microscopy, Biophotonics

## Abstract

Efforts to mitigate the COVID-19 crisis revealed that fast, accurate, and scalable testing is crucial for curbing the current impact and that of future pandemics. We propose an optical method for directly imaging unlabeled viral particles and using deep learning for detection and classification. An ultrasensitive interferometric method was used to image four virus types with nanoscale optical path-length sensitivity. Pairing these data with fluorescence images for ground truth, we trained semantic segmentation models based on U-Net, a particular type of convolutional neural network. The trained network was applied to classify the viruses from the interferometric images only, containing simultaneously SARS-CoV-2, H1N1 (influenza-A virus), HAdV (adenovirus), and ZIKV (Zika virus). Remarkably, due to the nanoscale sensitivity in the input data, the neural network was able to identify SARS-CoV-2 vs. the other viruses with 96% accuracy. The inference time for each image is 60 ms, on a common graphic-processing unit. This approach of directly imaging unlabeled viral particles may provide an extremely fast test, of less than a minute per patient. As the imaging instrument operates on regular glass slides, we envision this method as potentially testing on patient breath condensates. The necessary high throughput can be achieved by translating concepts from digital pathology, where a microscope can scan hundreds of slides automatically.

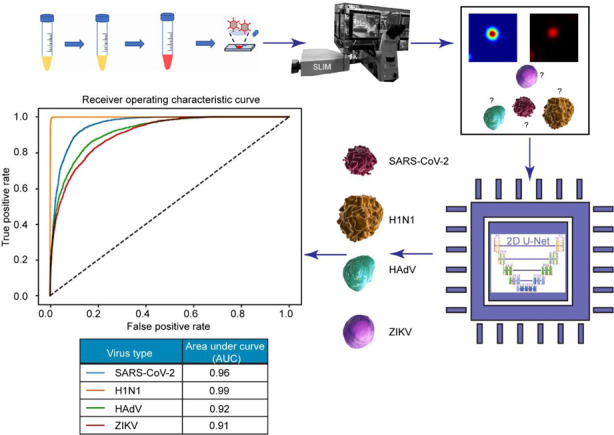

## Abstract (web summary)

Rapid label-free detection of SARS-CoV-2 using phase imaging (spatial light-interference microscopy (SLIM)) with computational specificity. Different virus types captured in SLIM image are detected and classified by 2D U-Net.

## Introduction

COVID-19 is an infectious disease caused by the severe acute respiratory syndrome coronavirus 2 (SARS-CoV-2), which reached pandemic proportions in 2020^1^. The global impact of the disease on the healthcare systems and its socioeconomic ramifications are severe and, likely, long-lasting^2^. The prompt response and public health measures have proven effective in limiting the spread of the virus, decreasing the number of active cases, and ultimately the mortality rate^3^. Fast, accurate, and scalable testing has been recognized unanimously as crucial for mitigating the impact of COVID-19 and future pandemics^4^.

Diagnostic test accuracy is characterized by the sensitivity, defined as the probability of a positive result in a diseased patient, and specificity, given by the probability of a negative result in a healthy patient. Furthermore, the negative predictive value represents the chance of an individual with a negative test to be disease-free and, conversely, the positive predictive value is the chance that a person with a positive test is infected. In addition to these accuracy metrics, throughput and cost are important for deploying testing at scale. Recently, Weissleder et al. have reviewed the current status of the COVID-19 diagnostic tests^4^. Briefly, nucleic acid tests (NATs) rely on the viral RNA being amplified via polymerase chain reaction (PCR) and are the most broadly used in the clinic today. NATs have been implemented on automated instruments and provide a result in several hours. Their accuracy may vary, with false-negative rates reported in the order of 30%^4,5^. Serological tests assess the patient’s response to the viral infection through proteins such as immunoglobulin G. The efficacy of these tests relies on prior knowledge about the patient’s immune status as well as potential previous exposures to other virus types. The accuracy of serological tests is very high when performed ~20 days after the infection or first symptoms, but may lead to high false-negative rates for early patients and false positives for patients previously exposed to other viruses^4^. Common antigen tests can be performed using nasopharyngeal swabs and yield results in less than one hour. These tests operate on detecting proteins associated with the SARS-CoV-2 virus (nucleocapsid or spike proteins) using lateral flow or enzyme-linked immunosorbent assay (ELISA) tests.

Recently, accelerated efforts have been devoted to developing alternative testing procedures. These alternative detection schemes involve the use of plasmonic biosensors^6–8^, fluorescence imaging of labeled virus particles and detection through machine learning^9^, microfluidic immunoassays coupled with fluorescence detections^10^, etc. While these approaches represent advances in SARS-CoV-2 detection methodologies, they still require either labeling or addition of foreign particles/solutions for the detection of SARS-CoV-2.

Holographic quantitative-phase imaging methods have been used for virus sensing and counting of HSV particles^11^. This study is an important precursor of our method and shares important similarities as well as differences. While both are quantitative-phase imaging techniques, methods of bringing specificity are different. Reference 11 uses chemical specificity based on antibody–antigen-based binding of virus particles on the glass slide. Virus particles are then counted based on their size estimate through computational means. In our study, for the generation of ground-truth data, we used fluorescence-stained virus particles and then used deep neural network for detection and classification of different virus types present in the sample. Other label-free imaging modalities, such as interferometric scattering microscopy (iSCAT)^12,13^ have shown tremendous potential for the detection of diffraction-limited samples, like nanoparticles^14^ and viruses^15–18^.

Here, we present a new approach for SARS-CoV-2 detection, which relies on direct, label-free imaging of viral particles. We employed spatial light-interference microscopy (SLIM), a highly sensitive interferometric method, to image viruses deposited on a glass slide. Although individual viruses are below the diffraction limit of the microscope, the optical path-length information retrieved by SLIM unravels the nanoscale distribution of the refractive index associated with the individual and aggregated viral particles. We paired these data with deep-learning algorithms, specifically optimized for viral-particle detection and classification. Using fluorescence markers for specific virus tagging, we retrieved “ground truth” data by imaging the same field of view with both SLIM and epifluorescence. To emulate a more realistic application environment, we synthesized datasets where different virus types were “digitally mixed” onto the same SLIM image for deep-learning development and evaluation. Thus, in addition to SARS-CoV-2, we imaged H1N1, HAdV, and ZIKV. While a situation where a patient is exposed simultaneously to these four viruses is highly unlikely, we wanted to test it as a challenging task for our method and evaluate the specificity of our deep-learning model. Following the training process, we tested the convolutional neural network (CNN) on unseen samples, classifying one virus type vs. the rest. Our results indicated a 96% area under the receiver-operating characteristic curve for SARS-CoV-2, 99% for H1N1, 92% for HAdV, and 91% for ZIKV.

This preclinical study demonstrates that sensitive imaging of unlabeled particles, paired with artificial intelligence (AI), can provide the foundation for a rapid, high-throughput, scalable test. The fact that the assay can be performed on the specimen placed on a glass slide allows for simple and fast sample collection, via, e.g., breath condensates. The image acquisition and inference take 100 ms in total, which means that the entire test, including specimen collection, can be performed within a minute. Throughput can be scaled up by borrowing engineering concepts from whole-slide scanners in digital pathology, where hundreds of slides can be automatically fed into the imaging instrument. As the specimen requires minimum preparation and the instrument can be made portable, in principle, the technology can be deployed as a point-of-care solution.

The paper is structured as follows. First, we present the workflow for multimodal imaging and ground-truth data acquisition. Next, we describe the SLIM imaging system and its sensitivity to the nanoscale ultrastructure of viral particles. We show 3D tomograms of the four virus types, to illustrate the subtle texture difference that the instrument captures, which the AI tools exploit for classification. We describe the convolutional neural network, which is a version of U-Net optimized for this problem. Finally, we present the accuracy of classifying the four virus types. We end with a discussion of the next steps necessary to implement this technology as a reliable clinical testing solution.

## Results

### Workflow

**Fig. 1:**
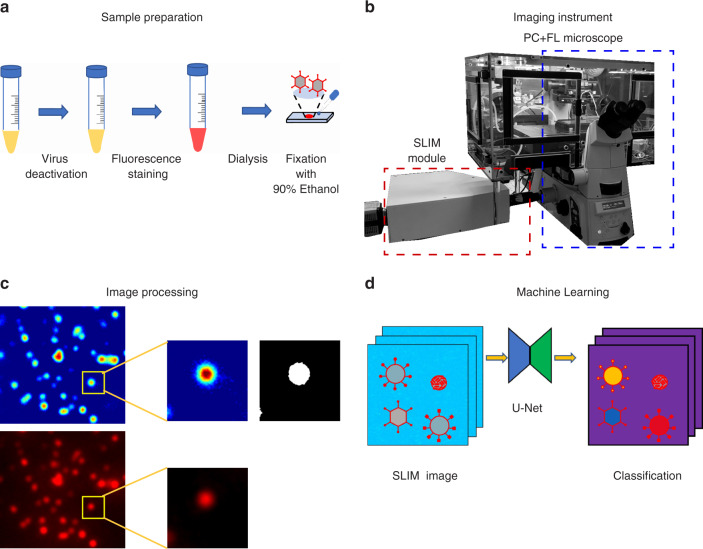
Virus-particle classification using SLIM and machine learning. **a** Sample-preparation protocol, viruses were deactivated, stained with Rhodamine B isothiocyanate, and dialyzed for two days to reduce fluorescence background, and then placed on a slide, fixed with 90% EtOH, and air-dried. **b** We added a SLIM module to a traditional phase-contrast microscope for quantitative-phase information. **c** SLIM and fluorescence were registered, single 48 × 48 spots were cropped from the image and segmented to provide a label for multiclass classification. **d** We synthesized a new dataset by randomly placing the cropped virus particles onto a background image acquired during the same experiment. A deep neural network was trained with this dataset to perform virus-particle classification. Given a SLIM image, the model will output a class label for each pixel in the image

Figure 1 depicts the workflow of our approach (see Fig. S1 and Supplementary Information Section S1 for details on sample preparation). We tagged the deactivated virus samples with Rhodamine B isothiocyanate as detailed in “Materials and Methods”. The staining was followed by dialysis to remove unbound fluorophores. The sample was deposited on a glass slide, fixed with EtOH, and air-dried (Fig. 1a). The slide was imaged using multimodal SLIM and epifluorescence, overlaid for the same field of view (Fig. 1b). The resulting images were processed to extract pairs of images associated with individual particles (Fig. 1c). A U-Net convolutional neural network was trained using these data, with the fluorescence images acting as ground truth. The U-Net output provides a semantic segmentation map, i.e., an image that classifies and labels the various virus types (Fig. 1d).

### Imaging procedure

**Fig. 2:**
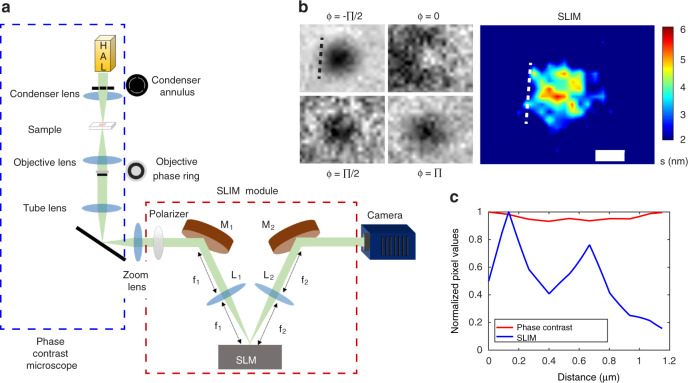
SLIM. **a** Optical configuration of SLIM. **b** Image reconstruction, with color bar representing optical path length (s), in nm. **c** Profile through yellow dotted line in **b**, showing high sensitivity of SLIM over phase contrast

A key element in our approach is the spatial light-interference microscope described in Fig. 2a. SLIM belongs to the family of quantitative-phase-imaging (QPI) instruments^19^, which have found broad applications in biomedicine^20–31^ due to their ability to image unlabeled, highly transparent structures. SLIM is implemented as an add-on module to an existing phase-contrast microscope and, in essence, controls rigorously the phase shift between the incident and scattered field emerging from the specimen^32,33^. We used a Nikon Eclipse Ti-inverted microscope outfitted with a SLIM module (CellVista SLIM Pro, Phi Optics, Inc.), which allows for fully automated data acquisition. The microscope objective pupil is relayed onto the surface of a phase-only spatial-light modulator (SLM), such that the phase shift between the incident and scattered light is controlled precisely (Fig. 2a). We record four intensity frames associated with individual phase shifts, applied in increments of π⁄2, as shown in Fig. 2b. The four intensity images are combined as described in^33,34^ to decouple the amplitudes of the incident and the scattered fields from the phase information and obtain a quantitative phase map associated with the specimen (Fig. 2b). Because the interfering fields in SLIM propagate along a common path, the phase measurement is highly stable to within a fraction of a nanometer pathlength^33^. Due to the white-light illumination associated with the phase-contrast microscope, the SLIM images are free of speckles, which convert into subnanometer spatial pathlength sensitivity^33^. These attributes make SLIM ideal for the challenging task of imaging viral particles on a glass slide. Figure 2c illustrates the significant boost in contrast present in SLIM compared with traditional phase-contrast microscopy.

To further investigate the surface morphology of SARS-CoV-2 particles captured in Fig. 2b, we imaged 200-nm polystyrene beads through SLIM. The surface roughness of polystyrene beads and SARS-CoV-2 particle displays significant differences as observed in Fig. S9. Furthermore, we also tested the spatial optical path-length sensitivity of our SLIM system by imaging sample-less area of a glass slide. Following the noise analysis in literature^33^, we measured the spatial optical path-length sensitivity to be 0.7 nm (see Supplementary Information section S5 and Fig. S10) which agrees well with the reported values in the literature^33^. To illustrate the detection of the ultrastructure in SLIM images, we simulated a model of two sub-diffraction-sized cylindrical particles, modeling spikes on the surface of SARS-CoV-2 particle. As discussed in Supplementary Information Section S6 and Fig. S11, while not resolved according to the Rayleigh criterion, the nanoscale profile can be detected by SLIM.

### Virus detection and classification via SLIM

**Fig. 3:**
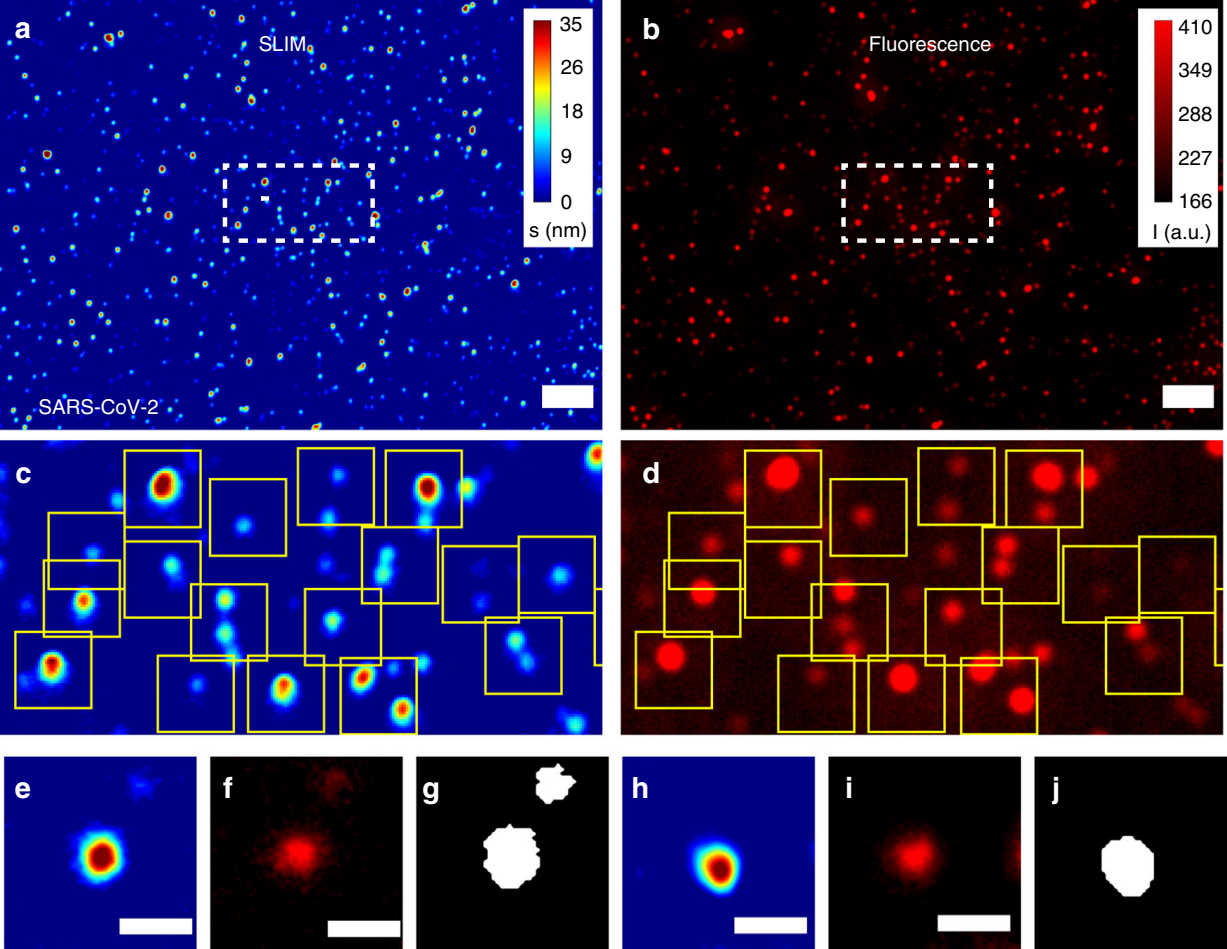
Correlated SLIM-fluorescence imaging results for SARS-CoV-2. **a** SLIM, colorbar represents optical path-length fluctuations in nm, and **b** fluorescence image, colorbar represents intensity in a.u., for the same field of view. **c, d** Cropped SLIM and fluorescence images from the region inside the white rectangle in **a** and **b**, yellow boxes highlight the correspondence between SLIM and fluorescence. **e** One 48 × 48 cropped image of SLIM, **f** fluorescence, and **g** corresponding segmentation mask prepared for AI. Another cropped set for **h** SLIM, **i** fluorescence, and **j** segmentation mask. Scale bar represents 5μm for **a, b** and 1 μm for **e–j**

SARS-CoV-2, H1N1, HAdV and ZIKV were separately stained as illustrated in Fig. S1 (see Methods section and Supplementary Information Section S1 for more details) with Rhodamine B isothiocyanate that has an emission at 595 nm. We performed dual-channel phase-fluorescence imaging on the samples. Figure 3 illustrates the imaging results for SARS-CoV-2, with SLIM (Fig. 3a) and fluorescence (Fig. 3b) images obtained on the same field of view. We registered the dual-channel images using MATLAB for perfect overlay (see Supplementary Information Section S2 for details on image acquisition and processing). The regions denoted by the dash rectangular selections in Fig. 3(a, b) are zoomed in and shown in Fig. 3(c, d). The discrete particles shown in the yellow rectangles reveal a 100% correspondence between phase and fluorescence, proving that SLIM is sensitive to the refractive index of the viral particles.

For machine learning, we cropped out single particles within 48 × 48 pixel images. Figure 3 (e–j) shows two examples of the cropped image set comprising of SLIM (Fig. 3(e, h)), fluorescence (Fig. 3(f, i)), and binary mask (Fig. 3(g, j)). Following the same imaging procedure, we imaged H1N1 (Fig. S2), HAdV (Fig. S3), and ZIKV (Fig. S4) for SLIM and fluorescence. We cropped out 48 × 48 pixel images and performed segmentation to produce labels for four classes of virus. Although our images are still diffraction-limited, SLIM’s nanoscale sensitivity to pathlength allows for efficient detection of viral particles.

Some signatures of the ultrastructure present in our SLIM data are demonstrated in deconvolved images (Supplementary Information Section S3). Using this operation, one can see that clumps of particles can be separated via deconvolution (Fig. S5).

### Deconvolution SLIM

Resolution of our imaging system is approximately 335 nm (illumination at 550 nm, objective 100x/1.45 with condenser NA 0.55). Following Rayleigh’s resolution criterion, two objects with separation less than the width of point spread function (PSF), cannot be fully resolved. The individual virus particles used in this study have an average diameter of less than 150 nm, which makes them subdiffraction objects for optical imaging. In order to push the resolution beyond the diffraction limit, we performed a deconvolution with the microscope’s PSF (Supplementary Information Section S3). To estimate the PSF, we identified the smallest spot in the images via a Matlab script. Using this PSF, the images were deblurred by employing the iterative Richardson–Lucy algorithm with total variation regularization (see Supplementary Information Section S3 for more details)^35,36^. Figure S5 illustrates the deconvolution results for the four virus classes. Thus, the deconvolution is able to produce deblurred images with clumps separated into smaller groups. However, it should be noted that the size of the deconvolved particles does not necessarily match the actual size of the virus particles as the decoupling of PSF and virus is still not perfect. However, we can successfully separate clumps into subsequent individual viruses, which the neural network is likely to pick up for classification.

### Quantitative analysis

One advantage of SLIM over fluorescence is the inherent ability to measure not only shape descriptors like diameter, orientation, circularity, etc., but also quantify the phase information associated with the sample, which can then be used to extract biophysical information, such as cell dry mass density. From the SLIM images, we extracted the total dry mass and surface dry mass density for each measured particle (see Supplementary Information Section S3 for details). We observed shifts in the dry mass density for different virus classes as shown in Fig. S6a. Figure S6(b–d) with *p*-values 1.35e-12, 8.84e-6 and 1.23e-5, respectively, demonstrates the statistical significance of the dry mass-density differences between SARS-CoV-2 and H1N1, HAdV, and ZIKV, respectively, obtained by applying Kruskal–Wallis test (in MATLAB) to single-virus data. These results indicate that dry mass density, which is incorporated into the SLIM data, is a marker that helps the machine-learning algorithm to detect SARS-CoV-2.

### Tomographic reconstructions

**Fig. 4:**
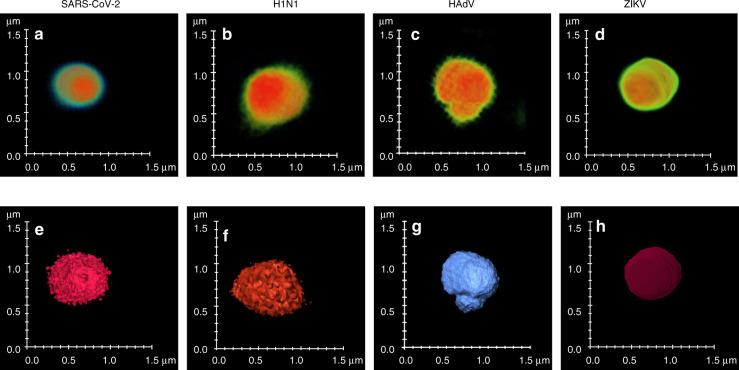
3D tomograms. Volume reconstruction of **a** SARS-CoV-2. **b** H1N1. **c** HAdV. **d** ZIKV. Surface reconstructions of **e** SARS-CoV-2. **f** H1N1. **g** HAdV. **h** ZIKV. All reconstructions were performed using the Amira software. Scalebars are representative of lateral dimensions of the respective particles

To get a better understanding of the viral particles, we performed a tomographic reconstruction of diffraction-limited SLIM, using the Amira (Thermo Scientific) software (see Supplementary Information Section S4 for details). The results are shown in Fig. 4, where volumetric reconstructions of the particle cores (Fig. 4 (a–d)), and surface reconstructions (Fig. 4 (e–h)) for each particle are illustrated. These reconstructions provide an insight into structural dissimilarities that exist even in the diffraction-limited SLIM images. Surface irregularities can be seen for SARS-CoV-2 in Fig. 4 (a,e). Figure 4 (b,f) shows the H1N1 particle, which again has an irregular surface but of different texture. Figure 4 (c,g) shows a clump of at least two HAdV particles with hexagonal boundary visible in the lower portion of Fig. 4g. ZIKV (Fig. 4 (d,h)) is significantly smoother compared with SARS-CoV-2. The structural signatures present in these reconstructions agree with the TEM images showing irregular surface morphology for SARS-CoV-2^37,38^ and H1N1^39^, hexagonal cross-section for HAdV^40^, and smoother surface of ZIKV^41,42^. These reconstructions suggest that signatures of structural information still exist in the diffraction-limited SLIM images, due to the nanoscale path-length sensitivity of SLIM. These subtle features help the machine-learning algorithm to successfully classify these particles.

To assess the structural differences on a large scale, we performed volumetric reconstructions of groups of particles. Movies S1–S4 show the overall structural differences in diffraction-limited SLIM images. It can be seen that the maximum-intensity projections of four virus classes exhibit differences in the structure, mainly, irregular surfaces for SARS-CoV-2 (Movie S1) and H1N1 (Movie S2), hexagonal projections for HAdV (Movie S3), and smoother surface for ZIKV particles (Movie S4). 3D surface reconstructions of a group of particles for each virus class are presented in Fig. S7.

The ultrastructure is less evident in the 2D images as its visibility is highly dependent on the orientation of the particle and focus during imaging. The effect of z-level slicing on the visibility of the ultrastructure is shown in Fig. S8, where z-levels marked by yellow boxes show higher visibility of surface roughness in the case of SARS-CoV-2 particle as compared with red-boxed image, where the virus core is in focus. Further evidence is shown in Supplementary video S5, which shows the 3D volumetric reconstruction for two SARS-CoV-2 particles.

### Convolution neural network

We formulated the virus-detection task as a semantic segmentation problem: given an input SLIM image containing several virus particles, our model predicts a probability distribution for each pixel, denoting the chance of this pixel belonging to one of the five classes: background, SARS-CoV-2, H1N1, HAdV, and ZIKV. An argmax operation turns the model output into a class label for each pixel. As all our raw SLIM images were of pure-culture virus particles, we synthesized a new dataset via “digital mixing” for machine-learning development and evaluation (see Supplementary Information Section S7 for details).

**Fig. 5:**
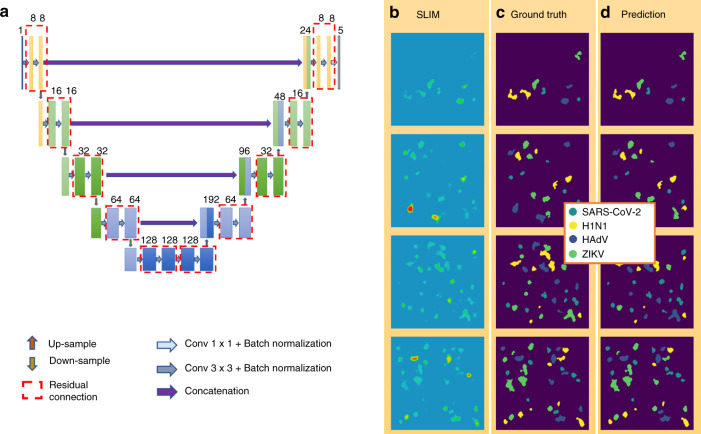
Training a deep neural network to perform classification of virus particles for the second dataset. **a** We used a modified version of U-Net for this semantic-segmentation task. Besides reducing the number of parameters in the network to around 0.8 million, we also added in residual connection and batch normalization for faster convergence. Model inference on images from the test set. **b** Synthesized images of mixed virus particles. **c** Ground truth label. **d** Model inference

The deep neural network we used was adapted from the U-Net (Fig. 5a and Fig. S12a)^43^. Our model was trained using the digitally mixed SLIM images as input and the corresponding segmentation maps as ground truth (Fig. 5(b,c) and Fig. S12(b,c)). We divided the machine-learning task into two steps. Two types of datasets were prepared based on two data-curation strategies. The first dataset was semiautomatic, with manual cropping followed by automatic segmentation, fixed concentration of viruses per digitally mixed image, and placement of virus particles on a grid with artificial-phase background. The second dataset was fully automatic, with automatic segmentation followed by automatic cropping, varying (but balanced) concentration of viruses per digitally mixed image, and random placement of virus particles on a blank image for digital mixing.

Our first model (Fig. S12) was a proof-of-concept test run. We manually cropped out 48 × 48 pixel regions of single virus particles from the images for all four viruses, collecting approximately 1200 cropped images. These cropped images were segmented, digitally mixed with an artificial background (see Methods section and Supplementary Information Sections S2 and S7). Every digitally mixed image has five particles per class. We kept 500 particles out as the test dataset, and trained the neural network on the remaining particles (see Supplementary Information Section S7). During evaluation, we noticed that our model sometimes predicted more than one label per particle. To solve this issue, we used a postprocessing strategy to enforce particle-level consistency in our model prediction (see Fig. S13 and Supplementary Information Section S7 for details on postprocessing method). After the postprocessing, we achieved the following area under the ROC curve (AUC) values for four viruses (Fig. S14a): 98% for SARS-CoV-2, 98% for H1N1, 96% for HAdV, and 97% for ZIKV. The average precision and recall for this model are 0.80 and 0.88 (SARS-CoV-2), and 0.82 and 0.73 (H1N1), 0.88 and 0.78 (HAdV), 0.82 and 0.84 (ZIKV) (see Fig. S14b).

**Fig. 6:**
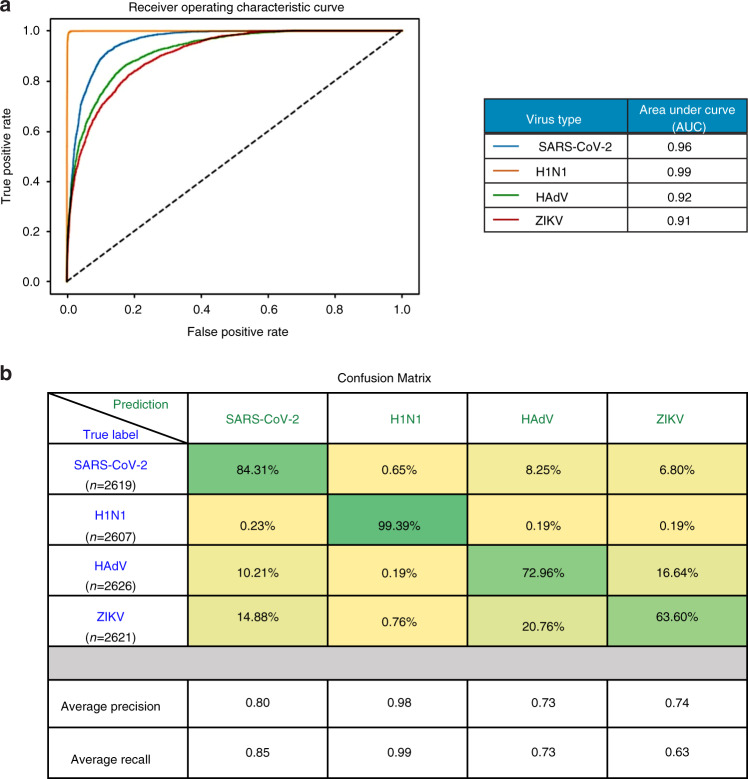
Model performance on the test dataset. **a** The receiver-operating characteristic (ROC) curve of the model on the test dataset. The model achieved over 0.9 area-under-curve (AUC) for all four virus types on the test dataset. The area-under-curve (AUC) for each class is computed by setting that class as label 1 and all other classes (the three remaining virus types) as label 0. **b** The confusion matrix of the model inference on the test dataset. Each row represents the ground-truth label, while each column represents the prediction. For visualization purposes, each entry in the confusion matrix was normalized with respect to the number of true labels (sum of each row). The precision and recall are averaged across all images in the test dataset. Both the ROC curve and the confusion matrix are evaluated on a per-particle level, where weighted average is computed to resolve conflict in model raw prediction

Our model’s excellent performance on this small, test-run dataset was the first step achieved in the direction of clinically usable, fast testing method. For the second phase of development, we moved on to a more realistic approach for data curation. To avoid bias in data selection and to focus on automation, we employed automatic processing to segment all the images and then crop out 48 × 48 particles from each image, based on the bounding box information of each particle, through a MATLAB script (see Supplementary Information Section S2). We emulated a real-life scenario where the concentration and position of particles per sample can vary. So, each image in our digitally mixed dataset had between 2 and 8 particles of each virus type, resulting in between 8 and 32 virus particles in total. In this dataset, all 4 types of virus particles were randomly placed onto over 1600, 240 × 240 blank (background removed by segmentation) images (see Supplementary Information Sections S2 and S7 for more details of the procedure). We randomly selected around 1000 images for training and kept the remaining 564 SLIM images as the test dataset to evaluate our model. Similar as the first dataset, we enforced instance-level consistency on our model prediction via the same postprocessing step (see Supplementary Information Section S7 and Fig. S13). Figure 5d shows the predictions after postprocessing. Quantitative results for this dataset are shown in Fig. 6, where Fig. 6a shows the one-versus-all receiver-operating characteristic (ROC) curve and Fig. 6b shows the complete confusion matrix to better illustrate our model’s sensitivity. AUC for all four virus classes is above 91%. We anticipate that, in clinical situations, the most challenging issue will be to detect the SARS-CoV-2 class alone, or, occasionally, distinguish it from the influenza virus (H1N1). The fact that the areas under the curve yield values of 96 and 99%, for SARS-CoV-2 and H1N1, respectively, is very encouraging. Average precision and recall values on the test dataset are 0.80 and 0.85 (SARS-CoV-2), 0.98 and 0.99 (H1N1), 0.73 and 0.73 (HAdV), and 0.74 and 0.63 (ZIKV) (Fig. 6b). We used gradient-weighted class-activation map (Grad-CAM) to visualize what regions of our SLIM images were crucial to the network’s performance in segmenting each type of virus particle^44,45^. Figure S17 indicates that the model paid uniform attention across the input image rather than focus more on the surface morphologies in ZIKV, which might help explain the relatively low performance on ZIKV particles.

We also plotted the precision and recall for SARS-CoV-2 on every image in the second test dataset into a histogram (Fig. S15). The majority of the detections have precision/recall values nearing unity. The learning-curve plots for both our models (for the first and second datasets) are shown in Fig. S16. The loss on the validation dataset and on the training dataset converged properly, indicating that our models did not overfit or underfit.

It is worth noting that our technique also works in case of virus aggregates. Figure S18 shows some of the model predictions for images that contain clusters, which are correctly classified. An important point to mention is that the introduction of any new class of particles will require us to retrain our model. To test the model performance in the case of dust or other subdiffraction, nonviral particles, we collected images of 25-nm polystyrene beads as a fifth class. We prepared a new dataset by combining four virus types and polystyrene-bead images. Following the same procedure as for the previous two datasets, we retrained a new network (Unet with efficientnetb0 as a pretrained encoder). Our model achieved 0.76 F-1 score for SARS-CoV-2 particles. The results for this training are shown in Fig. S19.

## Discussion

We presented a method for detection and classification of SARS-CoV-2 in the presence of other viruses, by using interferometric imaging and AI. Our results indicate that highly sensitive phase imaging is capable of providing subtle structural specificity of the viral particles, which in turn, allows for their accurate classification. There are two main components that help our model detect and classify viruses with high accuracy. First, the specific texture of the dry mass density can report on the differences in the refractive index caused by the specific protein compositions of the virus. Second, the nanostructure signature of individual viruses, e.g., irregularities on the surface of SARS-CoV-2 and H1N1, hexagonal shapes in HAdV, and the smoother surface of ZIKV, are subtle features in the SLIM images, exploited by the neural network.

The most likely combination of multiple viruses is SARS-CoV-2 and H1N1, a situation that can pose a challenge for accurate testing. However, our model proved to be successful in detecting and differentiating SARS-CoV-2 and H1N1 with a one-versus-all AUC of 96 and 99%, respectively.

We envision a COVID breath test based on the principle discussed in this paper, as shown in Fig. S20. A patient would exhale on a slide attached to a cloth mask. The slide would then be immediately taken for imaging. The clinical breath-condensate sample is expected to have other nonviral particles, such as bacteria, phospholipids, and aerosols containing potassium, calcium, and chloride, etc.^46,47^. These particles can be grouped into two categories according to their size as below and above the diffraction limit. Our model can successfully differentiate between dust particles and viruses as shown in Fig. S19. Particles bigger than 5 µm can be eliminated after prediction by threshold operation based on size of the particle. We chose 5 µm as a limiting size because viruses can form clusters with size less than 2 µm or greater than 2.5 µm^46^. Clusters can also be detected by our model as shown in Fig. S18. The limit of detection of our method is 1 particle in the largest field of view that the camera can capture as shown in Fig. S21. Assuming a frame size to be 2048 × 2048, the limit of detection turns out to be 1270 particles per cm^2^. In exhaled-breath condensate, the number of SARS-CoV-2 particles is estimated to be ~4000 particles per minute^48^. To capture virus particles on a premarked area of slide with dimensions 1 cm × 1 cm, we would require a minimum of ~1270 particles, which would require a minimum of 20 s of exhalation. However, our collection efficiency will be less than 100%, so we can increase the collection time to a few minutes as needed. Imaging prediction on an arbitrary field of view inside the 1 cm × 1 cm sample area takes less than a minute. This method will be very cost-effective, requiring only a common mask and a standard glass slide.

Pending successful clinical testing of this approach, we anticipate that the instrument can be implemented into a portable device controlled by a laptop. As the inference per field of view takes 60 ms, it is likely that the test per specimen, sampling several fields of view, will complete in a few seconds. Due to the lack of labels or other reagents, the test itself is bound to be inexpensive. Finally, to scale up throughput, we envision translating automatic slide-scanning engineering concept from digital pathology devices.

## Materials and methods

### Sample preparation

The viruses used in this study are: Heat-inactivated SARS-CoV-2 (ATCC^®^ VR-1986HK™), Influenza A virus (H1N1) (ATCC^®^ VR­-1894™), Human adenovirus 2 (HAdV) (ATCC^®^ VR-846™), and Zika (BEI: Zika Virus, PRVABC59, Infected Cell Lysate, Gamma-Irradiated (NR-50547)). HAdV and H1N1 were deactivated by UV. For fluorescence imaging, each virus solution was stained with Rhodamine B isothiocyanate, separately for each experiment. The Rhodamine B isothiocyanate (RBITC) can target any protein through the binding between isothiocyanate and amine group on the protein. Since the virus particles have a protein shell, it is effective to use RBITC to label them. Dialysis was carried out to remove unbound fluorophores from the stained solution. Stained virus sample was dropped on a glass slide, fixed with 90% ethyl alcohol, and air-dried (more information in Supplementary Information Section S1).

### Image acquisition and processing

We performed dual-channel correlative SLIM-fluorescence imaging on Nikon Eclipse Ti inverted microscope with add-on SLIM module (CellVista, Phi Optics, Inc.). Images were acquired with Nikon Plan-Apo 100x/1.45, phase-contrast oil objective. Exposure was kept at 30 ms and 200 ms for SLIM and fluorescence, respectively. For 3D reconstructions, we acquired a z-scan passing through focus, with a step size of 5 nm for the SLIM channel only. After the image acquisition, offline processing involved image registration of SLIM and fluorescence through MATLAB (see Supplementary Information Section S2). For the first dataset, we extracted 48 × 48 crops from SLIM and fluorescence images. We then segmented SLIM images to prepare the masks, which served as labels for the corresponding virus type during automated classification. For the second dataset, we first segmented the SLIM and fluorescence images and then performed automatic cropping based on bounding-box information (more information in Supplementary Information Section S2).

We performed deconvolution using Richardson–Lucy iterative algorithm with total variation (TV) regularization^35,36^. We first converted the phase map obtained from SLIM to complex field. This complex field was then used as an input to the algorithm. We derived an initial estimate for PSF from the images themselves, by choosing the smallest spot in the images. Utilizing the properties obtained from segmentation (area, integrated phase values, and centroid), we carried out quantitative analysis on single-virus particles using MATLAB (see Supplementary Information Section S3).

We produced tomographic reconstructions using Amira software (Thermo Scientific). We cropped out single particles from the whole image and upsampled them by a factor of 10 with bilinear interpolation to remove pixelations. We then used Volren and Isosurface rendering to reconstruct volume and surface tomograms (see Supplementary Information Section S4) for each virus type.

Calibration of spatial optical path-length sensitivity of our SLIM system was done by imaging sample-free area on a glass slide, as outlined in Supplementary Information Section S5. Our spatial optical path-length sensitivity was determined to be 0.7 nm.

### Machine learning

For both the first (manual selection with background) and second (automatic selection without background) datasets, we prepared digitally mixed images to train and test our network. We placed single-cropped viruses from each class, randomly in a 240 × 240 image, in fixed concentration for the first dataset (five particles per class) and varying concentrations (2–8 particles per class per image) for the second dataset. It is to be emphasized here that the digital mixing provides no distinction between the viruses, it only serves as a ground truth for model training. During training, the model weights were updated using the Adam optimizer^49^ against a categorical cross-entropy loss function. During evaluation, we found that in some cases, our model inferred more than 1 label for different parts of the same particle. To enforce instance-level consistency onto our model prediction, we performed a postprocessing step via connected-component analysis to ensure that all pixels in each individual particle are predicted as one class. After this postprocessing step (see Supplementary Information Section S7), our model’s performance was summarized into a confusion matrix on over 10,000 virus particles from the test dataset for the second dataset.

## Supplementary information

Supplementary Information

Movie S1

Movie S2

Movie S3

Movie S4

Movie S5

## Data Availability

All data required to reproduce the results can be obtained from the corresponding author upon a reasonable request.

## Code availability

All the code required to reproduce the results can be obtained from the corresponding author upon a reasonable request.
