# Supplementary material for: Label-free SARS-CoV-2 detection and classification using phase imaging with computational specificity
Source: Light Sci Appl. 2021 Sep 1;10:176. doi: 10.1038/s41377-021-00620-8 (PMC8408039; doi:10.1038/s41377-021-00620-8)
Supplement: Supplementary file 1 — Supplementary Information [file 41377_2021_620_MOESM1_ESM.docx]

**Supplementary Information for**

**Label-free SARS-CoV-2 Detection and Classification Using**

**Phase Imaging with Computational Specificity**

*Neha Goswami^a, c^, Yuchen R. He^b, c^, Yu-Heng Deng^d^, Chamteut Oh^e^, Nahil Sobh^c,f^, Enrique Valera^a,g,h^, Rashid Bashir^a,g,h,i,j^, Nahed Ismail^k^, Hyunjoon Kong^c, d^, Thanh H. Nguyen^e,i^, Catherine Best-Popescu^a,c^, Gabriel Popescu^*a,b, c^*

^a^Department of Bioengineering, University of Illinois Urbana-Champaign, Urbana, Illinois, 61801

^b^Department of Electrical and Computer Engineering, University of Illinois Urbana-Champaign, Urbana, Illinois, 61801

^c^Beckman Institute of Advanced Science and Technology, University of Illinois Urbana-Champaign, Urbana, Illinois, 61801

^d^Department of Chemical and Biomolecular Engineering, University of Illinois at Urbana-Champaign, Urbana, IL 61801, USA

^e^Department of Civil and Environmental Engineering, University of Illinois at Urbana-Champaign, Urbana, IL 61801, USA

^f^NCSA Center for Artificial Intelligence Innovation, University of Illinois at Urbana-Champaign, Urbana, IL 61801, USA

^g^Holonyak Micro and Nanotechnology Laboratory, University of Illinois at Urbana-Champaign, Urbana, Illinois 61801, United States

^h^Biomedical Research Center, Carle Foundation Hospital, 509 W University Ave., Urbana, Illinois 61801, USA

^i^Carle Illinois College of Medicine, 807 South Wright St., Urbana, Illinois 61801, USA

^j^Mayo-Illinois Alliance for Technology Based Healthcare, Urbana, Illinois 61801, United States

^k^Department of Pathology, College of Medicine, University of Illinois at Chicago, Chicago, IL, USA

* Gabriel Popescu

**Email:**  [gpopescu@illinois.edu](mailto:gpopescu@illinois.edu)

**S1. Sample preparation:**

**SARS-CoV-2:** Heat-inactivated SARS-CoV-2 was obtained from ATCC (ATCC® VR-1986HK™). The vial was centrifuged prior to opening. Since the virus was already heat deactivated, no further deactivation was performed. SARS-CoV-2 has a spherical structure with diameter ranging from 60-140 nm ^1,2^. The surface has spike like protein protrusions ^2^.

**Human adenovirus type 2 (HAdV)** was purchased from ATCC (ATCC® VR-846™). A549 cell line was used as host for HAdV, which was also obtained from ATCC (CCL-185). In brief, HAdV was propagated in A549 cells supplemented by Ham F-12 media with 2% fetal bovine serum (Thermo Fisher Scientific, MA, USA), and 1X antibiotic-antimycotic (Thermo Fisher Scientific, MA, USA). After 5 days of incubation at 37°C with 5% CO_2_ when 80% cytopathic effect was reached, HAdV was harvested from the cells by three freeze-thaw cycles. To purify HAdV, the virus solution was centrifuged at 2000 rpm (556 g) for 10 min (Sorvall Legend RT Plus, Thermo Fisher Scientific, MA, USA), and the virion-containing supernatant was filtered by a 0.45 μm membrane filter (Millipore Sigma, MA, USA). The virion-containing filtrate was then purified using ultracentrifuge with 36000 rpm (150700 g) at 4°C for 3 hours (Optima XPN-90 Ultracentrifuge, Beckman Coulter, CA, USA). The virus pellet on the ultracentrifuge tube was resuspended in 1X PBS and stored at -80°C before use. The infectivity of the purified HAdV was confirmed to be about 10^7^ PFU mL^-1^ by plaque assay.

The infectious HAdV was inactivated by ultraviolet (UV) irradiation at 254 nm. The UV irradiation at 254 nm was known to inactivate HAdV by primarily damaging genomic DNA ^3^. The UV irradiation at 254 nm was generated in this study using a medium-pressure UV generator (Calgon Carbon Co., Pittsburgh, PA) with a bandpass filter at 254 nm. We exposed 100 μL of virus solution to the UV irradiation (254 nm) for about 15 min which was equivalent to about 300 mJ cm^-2^. According to Ref. ^3^, the fluence of 300 mJ cm^-2^ was expected to inactivate HAdV by 10-log reduction. HAdV has an icosahedral shape, with diameter of about 100 nm ^4^.

**Zika Virus (ZIKV):** The Zika virus, PRVABC59 (BEI: Zika Virus, PRVABC59, Infected Cell Lysate, Gamma-Irradiated (NR-50547)) was gamma-irradiated (5 x 10^6^ RADs) on dry ice. The sample was diluted in Nuclease-free water. The ZIKV particles are spherical (approximately 50 nm in diameter) with a 30 nm electron-dense core ^5,6^. The mature ZIKV contains 180 copies of the envelope protein (E) and membrane (M) proteins in icosahedral-like organization and arranged in the raft configuration ^5,6^. The E protein predominates on the surface with the M protein residing underneath the E protein ^5^. The raft configuration consists of three E proteins dimers which lie parallel to one another, with the virion having a total of 30 rafts ^5^. ZIKV has an imperfect icosahedral structure with a diameter of approximately 50-60 nm ^7,8^.

**Influenza-A (H1N1)** was obtained from ATCC (ATCC® VR­1894™). UV irradiation with 254 nm wavelength was also applied to inactivate the viruses. Although UV irradiation could cause structural change in capsid proteins, the primary inactivation mechanism of the UV irradiation is known to mutagenize the genomes ^9^. The virus stock was used without neither further inoculation in cells nor the purification. The influenza virus was exposed to the same UV irradiation system described for HAdV inactivation, but the exposure time was 10 min which is equivalent to 200 mJ cm^-2^. This fluence was expected to inactivate the viruses about 30 log-reduction assuming the inactivation rate follows the first-order reaction ^10,11^. H1N1 is a spherically shaped particle with a diameter of approximately 100 nm, it also features spike like protrusions on the surface ^12^. However, it may also exhibit pleomorphism resulting in elongated structure ^12^.

**Fluorescence Tagging:** All four virus particles were tagged separately with fluorescent probes in order to validate their presence for observation. For each experiment, virus particles were suspended in 5 mL of carbonate/bicarbonate buffer (0.1 M, pH=9.2). Then, 1 mL of rhodamine B isothiocyanate (RBITC) (2 mg mL^-1^ in DMSO) solution was added into the virus solution under stirring condition. The RBITC binds onto virus through conjugation between isothiocyanate group and amine group on capsid of virus. The reaction was conducted for 2 hours and protected from light. In the end, the virus particles tagged with rhodamine B were purified using dialysis against deionized water. After 2 days, the purified virus particles tagged with rhodamine B solution was poured on glass slide for fixation with 90% Ethyl alcohol. Staining procedure is depicted in Figure S1.

**Slide preparation:** 10 µL of stained virus solution was dropped on a plain glass slide and allowed to air dry. Upon complete drying, the sample spot was treated with 90% Ethyl alcohol for fixation followed by air drying again. Completely dried sample was then covered with coverslip (#1) and the edges were sealed with nail polish.


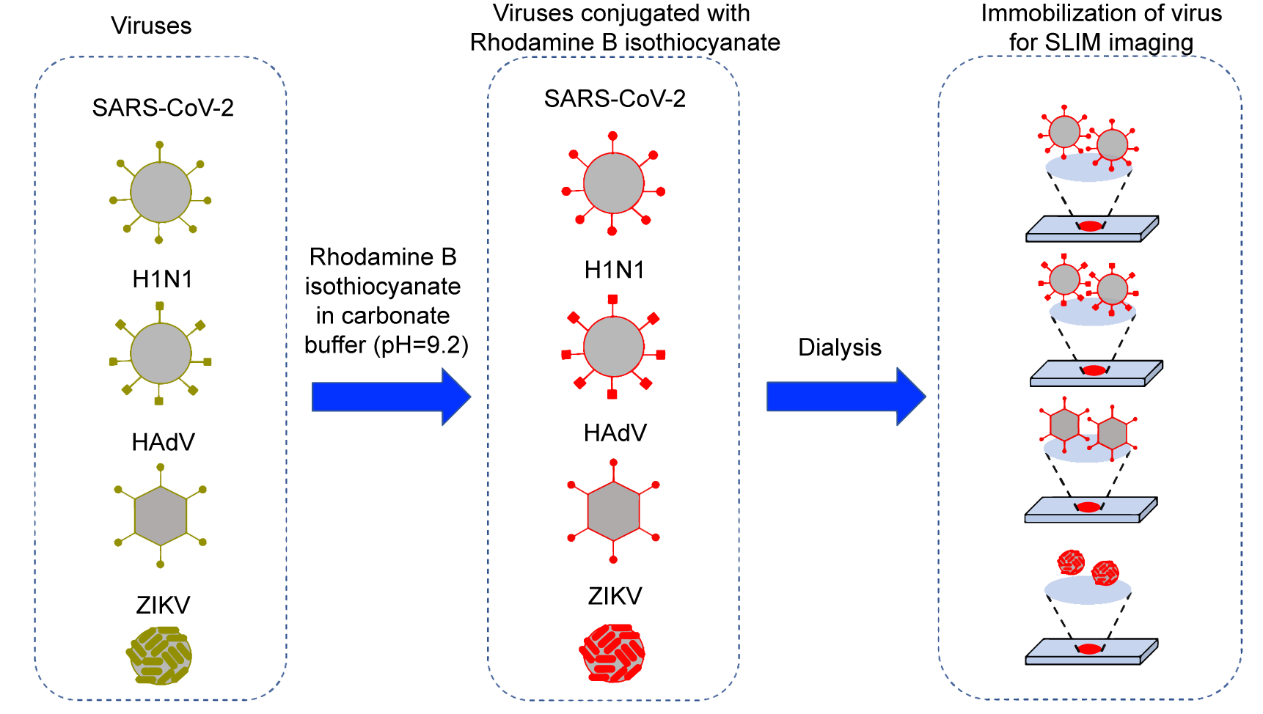


**Figure S1. Fluorescence staining and sample preparation process:** The process of fluorescent tagging for virus particles.

**S2. Image acquisition and processing: registration, cropping and segmentation**

**Image acquisition:** We performed dual channel phase-fluorescence imaging. Nikon Ti-E microscope was used for these experiments with SLIM module (CellVista SLIM Pro, Phi Optics, Inc.) connected to the left port of the microscope. SLIM acquisition was performed using Andor-Zyla camera. For the fluorescence, we used Zeiss AxioCam MRm camera mounted on the right port of the microscope. Imaging was done using Nikon Oil immersion 100x/1.45 phase contrast objective, with 18 pixels μm^-1^ resolution (including 1.2x magnification provided by SLIM module). For fluorescence, TRITC filter was used with exposure time 200 ms. For each field of view, we used CellVista software (Phi Optics, Inc.) to capture a pair of SLIM and fluorescence images. SLIM can produce images at 15 fps. Fluorescence was required for generating the ground-truth for machine learning on the phase data. For each field of view, synchronized and sequential acquisition was performed for the two channels. SLIM image was acquired on Andor-Zyla camera with a resolution of 1392 × 1040 pixels. Corresponding fluorescence images were captured by the Zeiss AxioCam MRm camera with an image size 1388 × 1040 pixels. Imaging results for SARS-CoV-2 are shown in the main text Figure 3.

Following the same procedure as outlined in main text, we imaged H1N1, HAdV and ZIKV. Figures S2-S4 represent the imaging results for H1N1, HAdV, and ZIKV, respectively. SLIM and fluorescence images for the same FOVs are shown in subfigures a and b, with 48 x 48 pixel SLIM, fluorescence, and SLIM mask, respectively, shown in c, d and e.


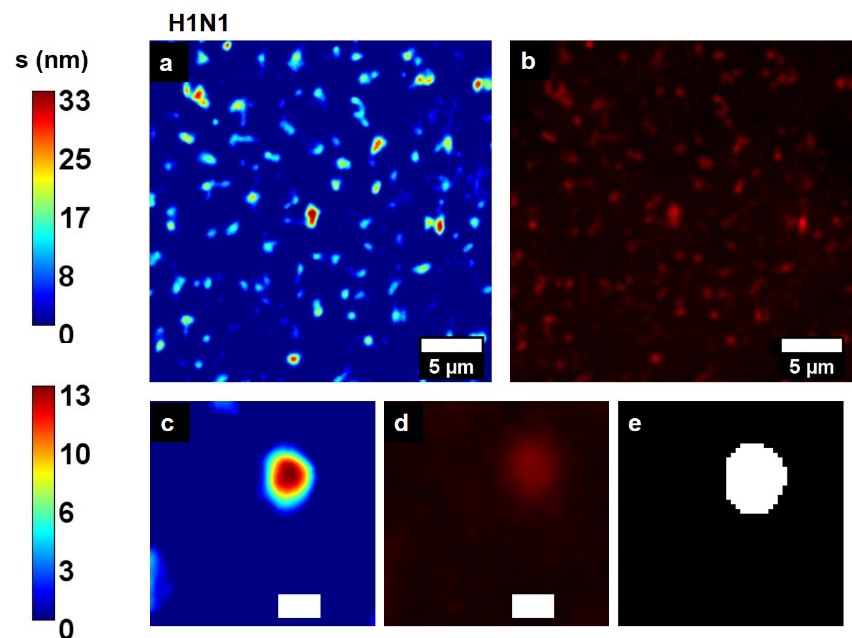


**Figure S2. Correlated SLIM-Fluorescence imaging results for H1N1 Virus:** a. SLIM b. fluorescence for the same field of view, colorbar representing optical path length fluctuations in nm. c, d, Cropped single virus spots for SLIM and fluorescence, respectively. e. SLIM mask for AI training. Scale bar represents 5 μm for a, b and 0.5 μm for c, d.


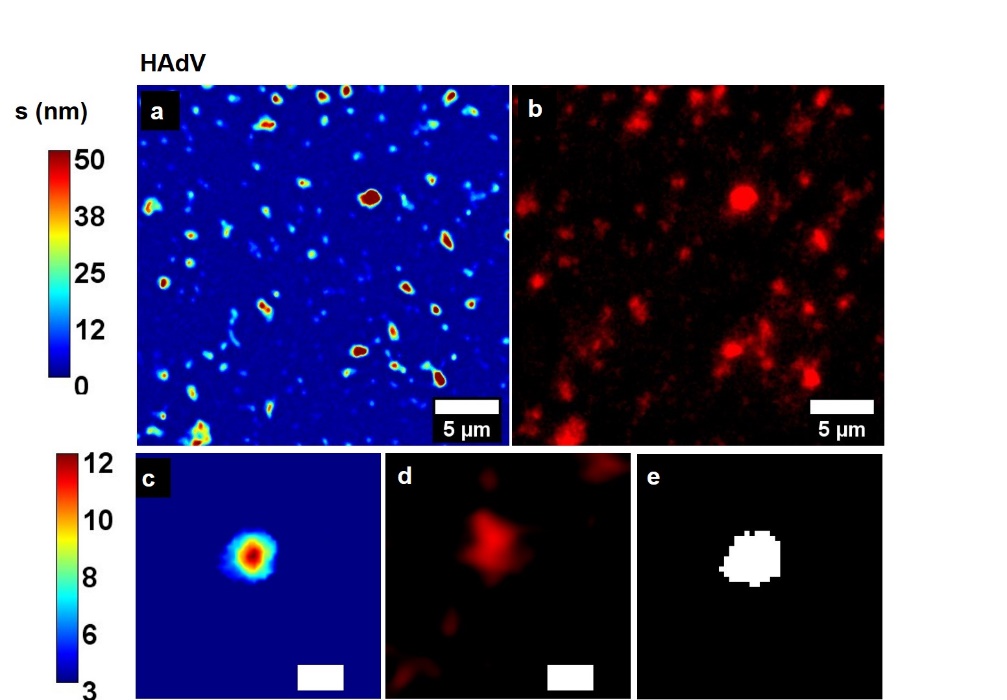


**Figure S3. Correlated SLIM-Fluorescence imaging results for HAdV:** a. Phase map obtained from SLIM, colorbar representing optical path length fluctuations in nm b. Fluorescence image for same field of view. c and d represent an example of one cropped virus particle (48 x 48 pixels) for SLIM and fluorescence respectively, with e representing the segmentation mask for labelling. Scalebar: 5 µm for a, b and 0.5 μm for c, d.

Image overlay involved two sequential image registrations. MATLAB scripts were used for both the steps of registration. First registration was based on manual control point selection and similarity transformation. For the second step, the resultant transformed image was then used as a moving image in MATLAB app called Registration Estimator, using a ‘multimodal’ registration with translation.


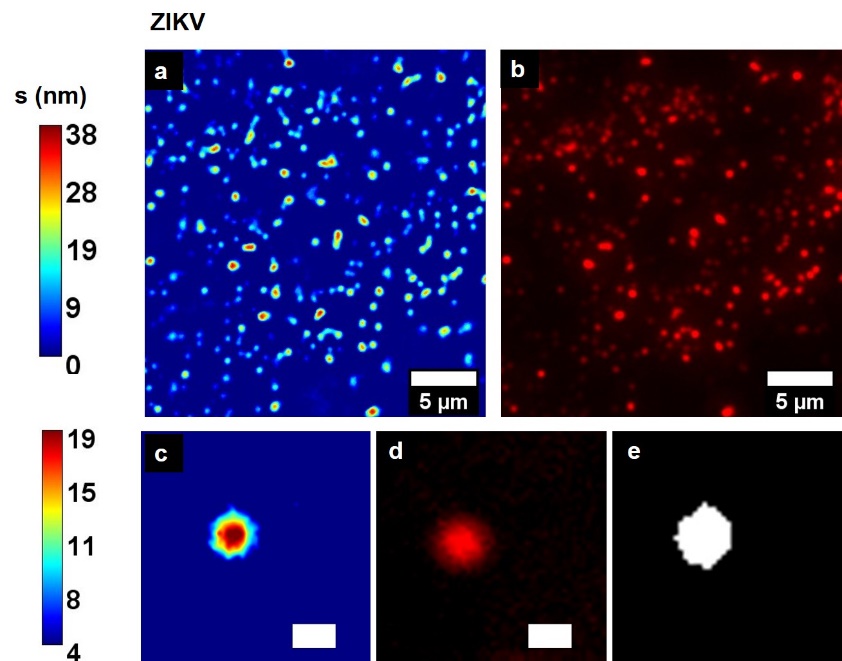


**Figure S4. Correlated SLIM-Fluorescence imaging results for ZIKV:** a. SLIM b. fluorescence for the same field of view, colorbar representing optical path length fluctuations in nm. c, d, Cropped single virus spots for SLIM and fluorescence, respectively. e. SLIM mask for AI training. Scale bar represents 5 μm for a, b and 0.5 μm c, d.

For the first, test-run dataset, we manually selected 48 × 48 cropped images from both SLIM and fluorescence images using a macro written in FIJI (ImageJ) that synchronizes the coordinates of ROI selection for both images of same FOV. These cropped SLIM images were then segmented to be used as labels for machine learning algorithm. Care was taken to select single particle and avoid clumps.

For the second, main dataset, we prepared binary masks from the SLIM images. For training the neural network, 48 × 48 pixels cropped images were prepared based on bounding box information from ‘regionprops’ function (MATLAB). These masks would serve as a label for the four classes. We carried out segmentation using scripts written in MATLAB. Input images were median filtered with a neighborhood window of 5 pixels to reduce noise. Adaptive thresholding was performed with a sensitivity of 0.65 and a neighborhood size of 17 pixels in each dimension. Morphological operations like open, fill and dilate were used to refine the mask and remove stray elements. Considering 335 nm as our minimum detection spot width based on our system PSF, anything with area less than 29-pixel square was not selected in the final mask. Finally, to remove separate objects with borders touching, we used distance transformation and watershed algorithm. Same parameters were used to segment all four-virus dataset to avoid bias. For each detection, a label was created and ‘regionprops’ function (MATLAB) was used to measure all properties. These properties were used to extract sum of phase values over the area, equivalent diameter, centroid, circularity, bounding box coordinates and pixel index of the area to be used for quantitative analysis to calculate dry mass, dry mass density and selection of PSF estimate (through equivalent diameter and circularity). We imaged 13,143 SARS-CoV-2, 18,763 H1N1, 9,346 HAdV, and 12,299 ZIKV particles. The lower number of detections in HAdV can be attributed to the clumping nature that we observed in the sample.

We also prepared fluorescence masks to validate our SLIM selections. The segmentation procedure was the same as for SLIM. However, for the adaptive thresholding, we chose sensitivity to be 0.55, with a neighborhood size of 27 pixels along each dimension.

**S3. Deconvolution and quantitative analysis:** For the deconvolution, the smallest spot in the images was identified through a script in MATLAB and assumed to be the initial estimate of the PSF. Using this PSF estimate for deconvolution, the deblurred image was obtained. Deconvolution was performed in MATLAB. We used iterative, Richardson-Lucy (RL) algorithm in conjunction with total variation (TV) regularizer ^13,14^.

The algorithm works on the complex field image, which is defined as

[1]

where *ϕ(x,y)* is the SLIM image. The algorithm iteratively solves for ^13,14^

$I^{m+1}(x,y)=A(x,y)\left[I_{psf}(-x,-y)\otimes\frac{I^{o}(x,y)}{I_{psf}(x,y)\otimes I^{m}(x,y)}\right]$ [2]

Where *I^0^* is the observed image, *I_psf_* is the estimate of PSF, and *A(x, y)* is the combination of the present image and Total Variation regularizer, given by ^13,14^

. $A(x,y)=\frac{I^{m}(x,y)}{1-\beta\nabla.\left(\frac{\nabla I^{m}(x,y)}{\left\|I^{m}(x,y)\right\|}\right)}$ [3]

In Eq. 3, *β* is regularization parameter. The iteration is terminated when there is no significant change between iterations. Finally, the phase is extracted from last iteration results to yield deconvolved SLIM.

[4]

Smallest detection in each dataset for all four-viruses was used as the initial estimate of PSF. To get this estimate, we selected particles with equivalent diameter less than 7 pixels (which translates to 388 nm, to cover our estimated width of PSF, 335 nm) and circularity greater than 0.8. Out of this selected set, we selected random samples of images based on visual feedback and applied ‘Smooth’ operation in FIJI (ImageJ) on these selected images to reduce spurious pixel noise. We generated surface plot for each of the selections and the images with multiple peaks were discarded. Final estimates were then tested on the deconvolution algorithm one by one to select the best estimate that would not introduce significant ringing, edge effects or noise. We note that although the deconvolution is not able to fully resolve the individual viruses, it still deblurs the images to show morphological evidence (hexagonal shape, in case of HAdV, Figure S5c) of the virus structure in the detected particles. Figure S5 shows the deconvolution results for all four viruses.


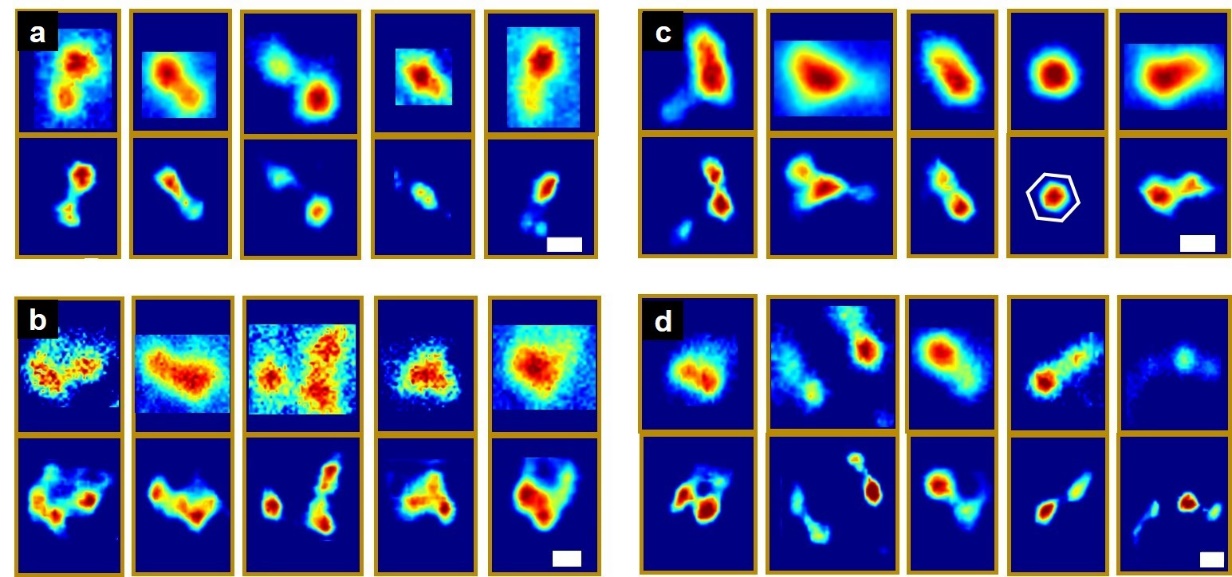


**Figure S5. Deconvolution results:** Inside each subfigure, raw SLIM images are in top row and deconvolved SLIM images are in bottom row for a. SARS-CoV-2 b. H1N1 c. HAdV, with hexagonal shape highlighted in 4^th^ example and d. ZIKV respectively. Scalebar is 0.5 μm for all images.

**Quantitative analysis: Dry mass, dry mass density**

Dry mass is a measure of non-aqueous mass of the biological sample ^15^. It is defined as ^15^

[5]

Dry mass density is the ratio of dry mass to area and is defined as

[6]

In Eq. 6, *λ* is the central wavelength of illumination, 550 nm, *η* is the refractive index increment with a value of 0.2mL g^-1^ ^15^ and *ϕ* is the phase shift introduced by the sample.

We calculated and compared dry mass density of carefully selected virus images, excluding large clumps. Figure S6a shows the histogram of dry mass density for all four viruses, which clearly shows the peak separations between the four classes. To investigate the statistical significance of these peak differences, we applied Kruskal-Wallis test (due to non-Normality of the data) in MATLAB on the dry mass densities to single virus data of all four virus classes. Results are shown in Figures S6b-d for dry mass density difference between single virus particle images selected by MATLAB script for SARS-CoV-2 and H1N1, HAdV and ZIKV respectively. The p-values are 1.35e-12, 8.84e-6 and 1.23e-5 for Figures S6b-d respectively showing the high significance of dry mass density difference of SARS-CoV-2 and other viruses in this study.


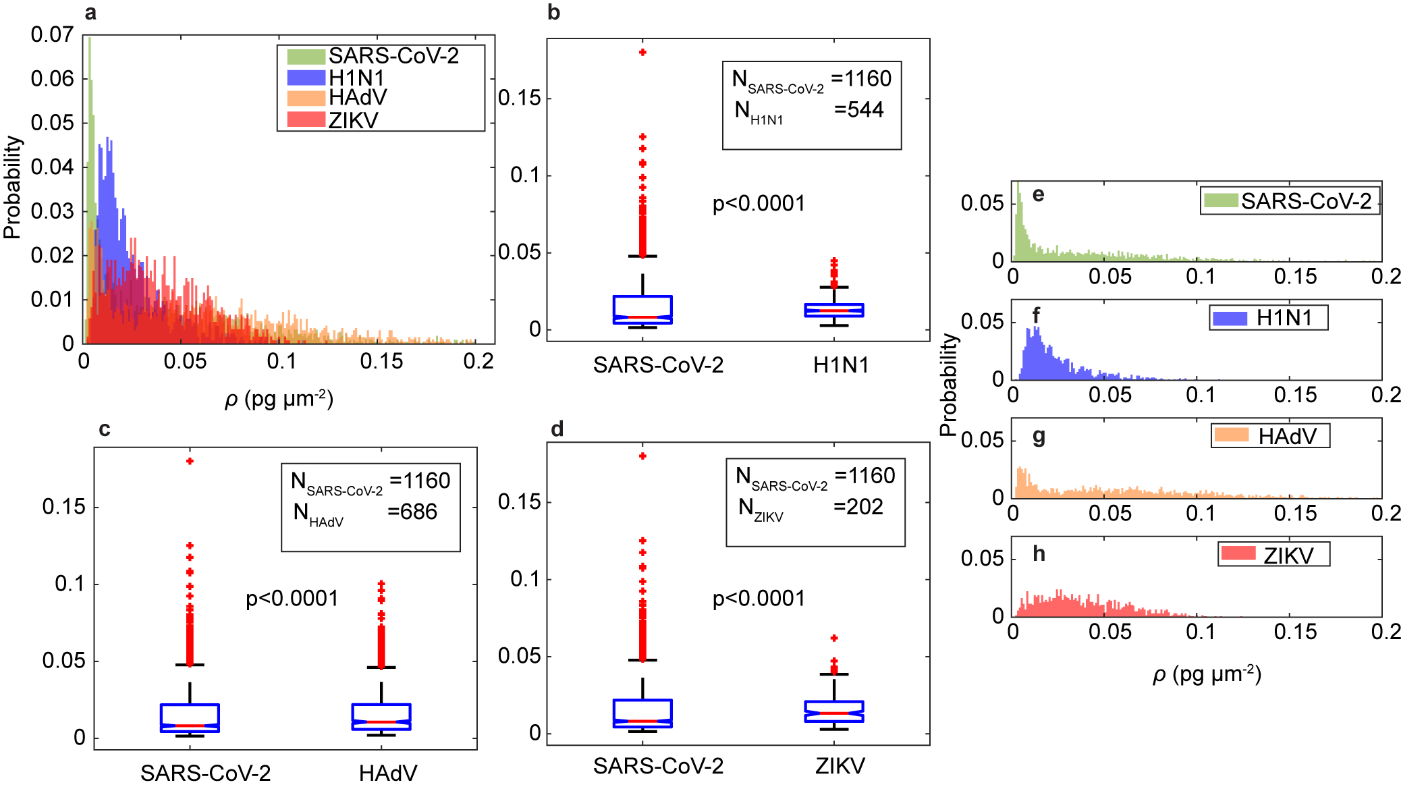


**Figure S6. Quantitative analysis: a.** Dry mass density histogram for all four viruses, showing distinct peak for SARS-CoV-2. **b, c, d.** Kruskal-Wallis test results for dry mass density differentiation between SARS-CoV-2 and H1N1, HAdV and ZIKV single virus particles respectively. p-value in all cases is <0.0001, indicating high significance. Number of particles in each test is mentioned on the graphs. Zoomed in histograms from a for **e.** SARS-CoV-2, **f.** H1N1, **g.** HAdV and **h**. ZIKV particles, respectively.

**S4. Tomographic reconstructions**

Motivated by the structural signatures provided by SLIM, we investigated the possibility of retrieving more information out of the 3D reconstructions from z-scan of SLIM images. For this purpose, we did a z-scan SLIM acquisition, ~2 μm above and below the focus of particles, with a step size of 1-5 nm, using a 100x/1.45 NA objective. Out of the z-stack, substack covering slices extending from just above to just below the surface of the virus particles was selected. For the reconstruction, we used the Amira (Thermo Scientific) software and segmented images either through MATLAB or through Amira’s functions, ‘labelfield’ and ‘segmentation’, depending on the accuracy of the resulting label. Images were resized by a factor of 10 and bilinear interpolation was used for better visualization. Volumetric rendering was performed in Amira, after a histogram equalization of intensity to enhance contrast. Isosurface rendering was performed to create surface rendering. Both the volumetric and surface reconstructions through Amira are shown in main text Figure 4, and Movies S1-S4. Figure S7 shows the 3D surface reconstructions of a group of virus particles.


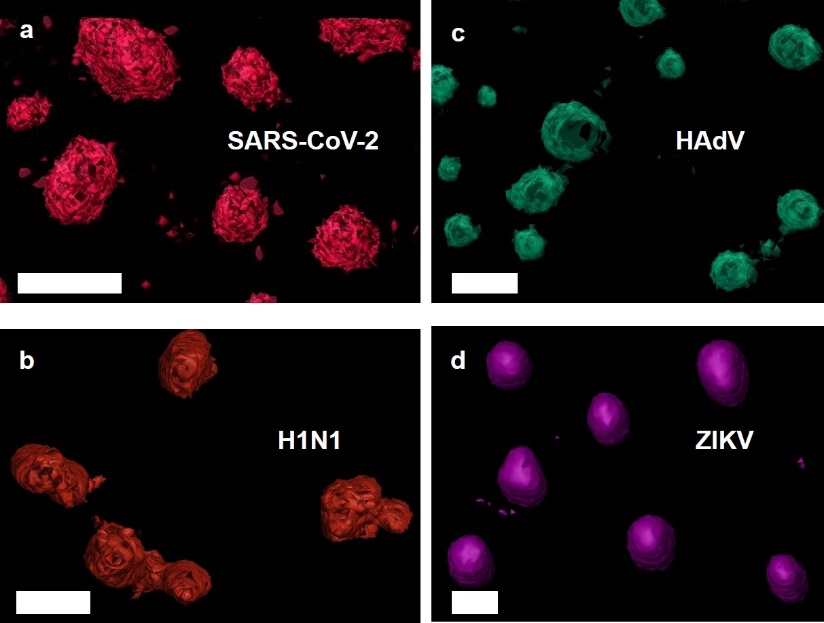


**Figure S7. 3D Surface reconstructions** of a SARS-CoV-2, b H1N1, c HAdV and d ZIKV particles. Scalebar: 1 µm

The ultrastructure observed in virus particles is highly dependent on the orientation and z-position of the image captured. Figure S8 and supplementary movie S5 show that during a z scan, some of the z-slices show higher evidence of ultrastructure (images within yellow boxes in Figure S8) than others where virus core is in-focus (image within red box in Figure S8).


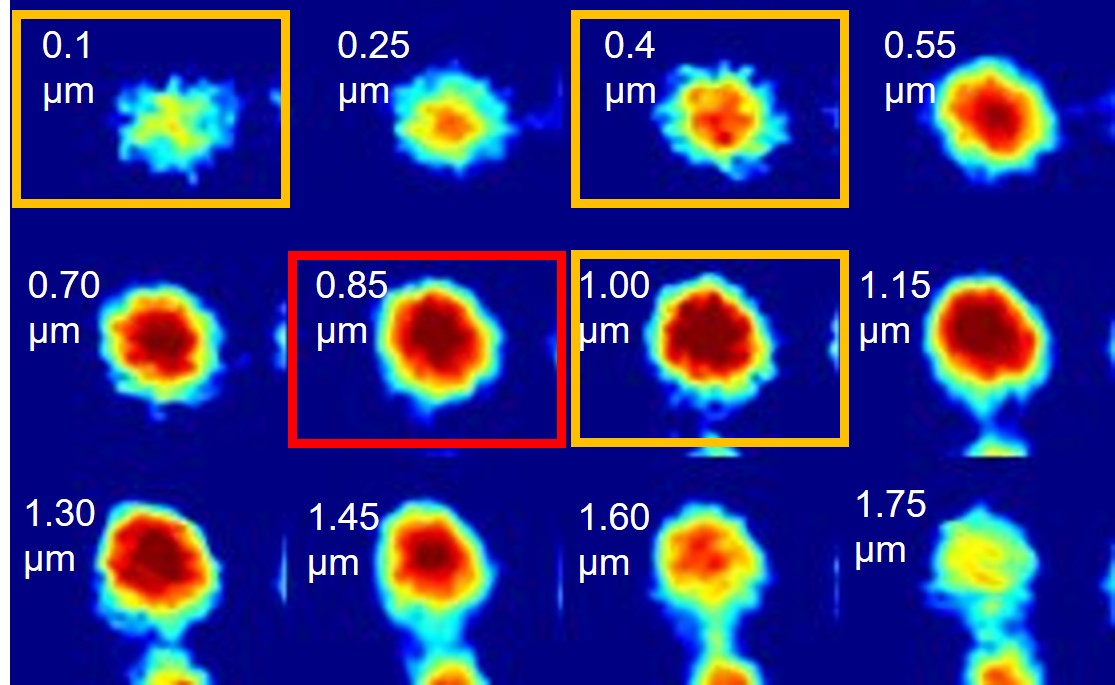


**Figure S8. Presence of nanostructures at different z-levels:** z-slices of SARS-CoV-2, showing evidence of nanostructures in yellow boxes. Red box highlights focused planes where nanostructures are less evident. Numbers denote z-slice level.

**S5. Calibration of phase sensitivity and noise:**

To validate that the nanostructures observed were not noise, we imaged 200 nm polystyrene beads with SLIM. Figure S9 shows that the surface irregularities display significant differences in the case of beads vs. SARS-CoV-2 particles.


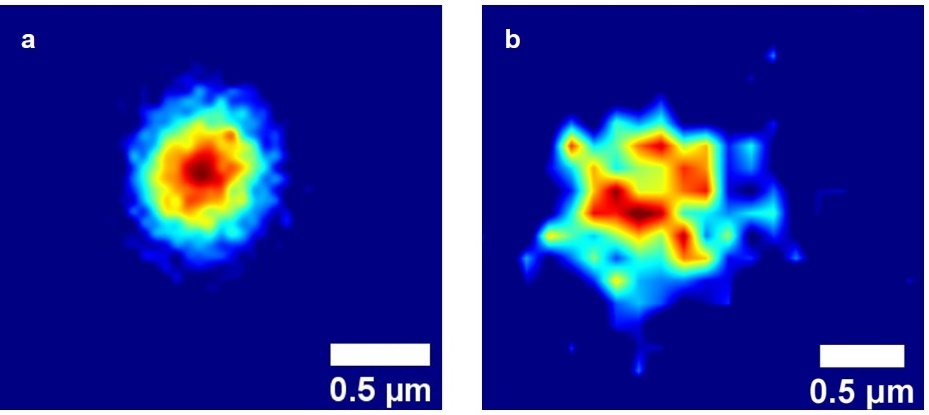


**Figure S9. Comparison of surface irregularities of a**. 200 nm polystyrene bead, **b.** SARS-CoV-2 particle.

To evaluate spatial optical path length sensitivity, we imaged a portion of glass slide where no sample was present. Figure S10a shows the SLIM image of a sample-less region with the histogram of optical pathlength shown in Figure S10b. A Gaussian fit of the histogram provides a standard deviation of 0.7 nm, which represents our spatial pathlength sensitivity.


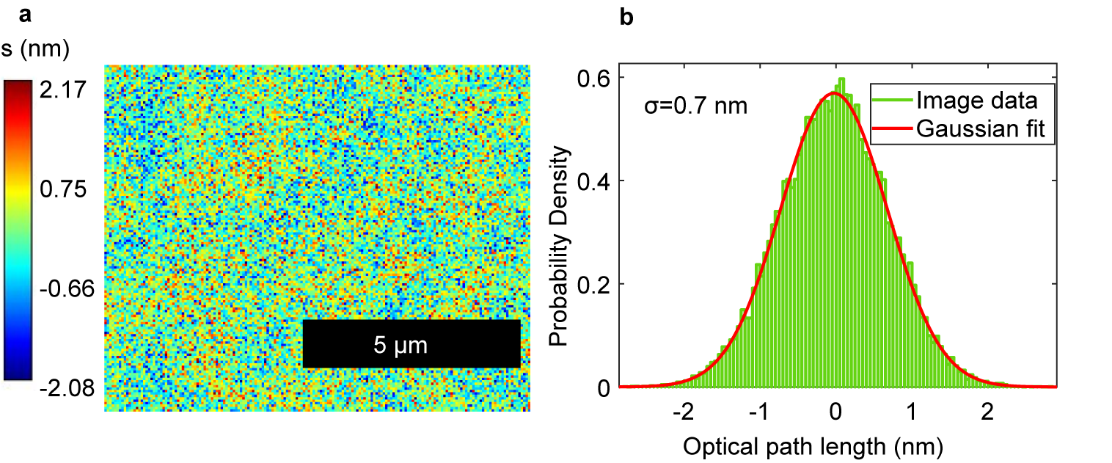


**Figure S10 Spatial pathlength sensitivity for the SLIM system**. a. SLIM image for a sample-less field of view with colorbar indicating the optical path length in nm. b. Histogram of the optical path length values (green) with a normal distribution fit (red), of standard deviation σ=0.7 nm, indicating the spatial pathlength sensitivity of the SLIM system.

**S6. Simulation of SARS-CoV-2 surface irregularities**

To validate the presence of nanostructures in SLIM images, we developed a simulation model comprising of two sub-diffraction cylinders of width ~120 nm, height 50 nm on the surface of a virus body of diameter 100 nm. These cylinders are separated by 200 nm, as shown in Figures S11a, d. The point spread function of the microscope is modelled to be of 350 nm width as shown in Figures S11b, e. The PSF-blurred image of the two cylinders is shown in Figure S11c. The optical path length profile of the outer surface of the blurred spot is shown in Figure S11f, where the profile shows two optical path length peaks. These peaks are not resolvable, however, they have amplitudes greater than the SLIM sensitivity, indicating that the ultrastructure can be captured through our SLIM system owing to the sub nm optical pathlength sensitivity.


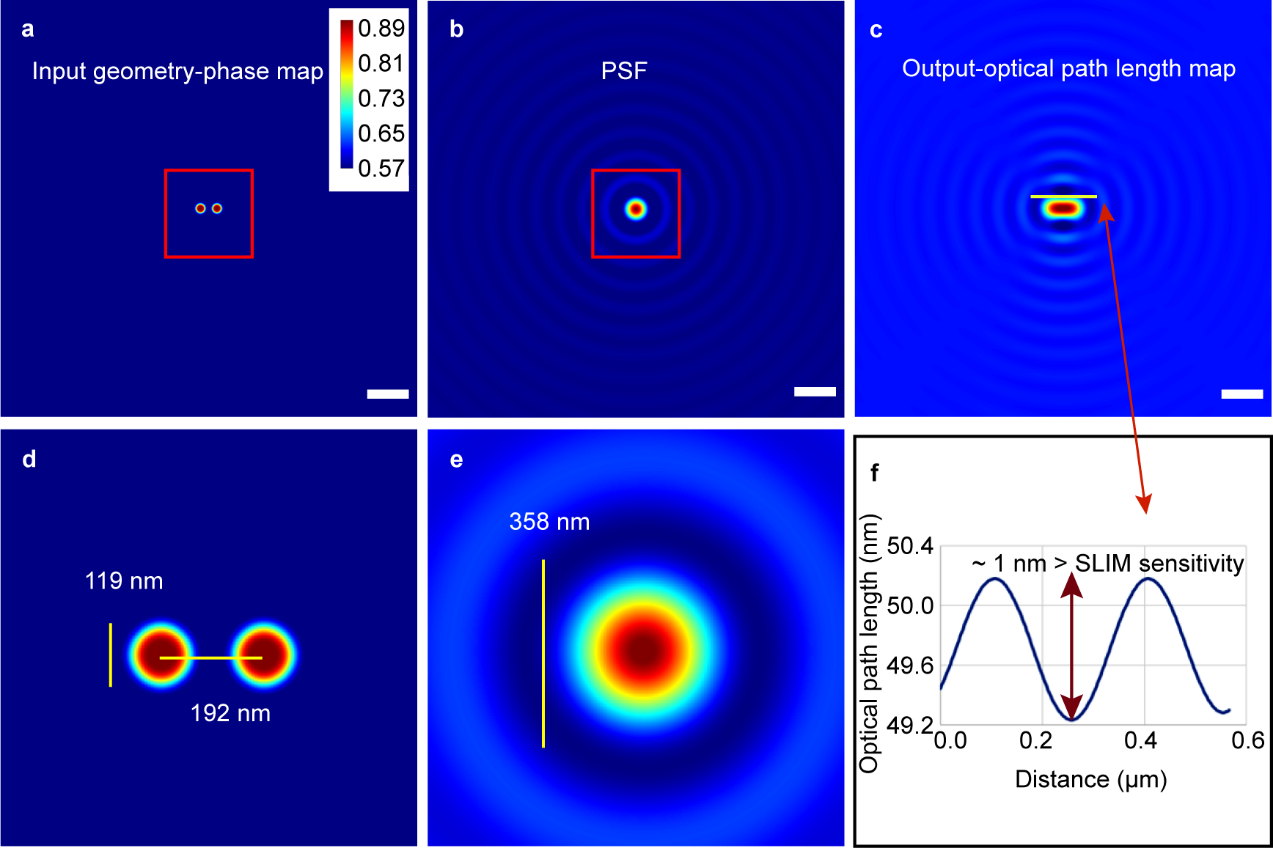


**Figure S11 Simulation results for ultrastructure visibility in SLIM**. a. SLIM image model for two cylinders representing surface irregularities of the SARS-CoV-2 virus, with the colorbar indicating the optical phase shift in radians b. Modelled PSF of the microscope. C. Optical path length map of the SLIM image blurred by the PSF, d. Dimensions of the cylinders inside the red box in a, e. Dimensions of the PSF inside the red box in b, f. Optical pathlength profile along the yellow line indicated in c, with the difference between the two spikes producing a significant change in optical path length, above the sensitivity of SLIM system. Scalebar is 0.5 µm for a, b and c.

**S7. Machine learning: Development of deep learning models**

**Digital Mixing:** For the training and subsequent test and validation of our network, we employed a scheme called digital mixing. 48 × 48 cropped images of all four detected virus particles (SARS-CoV-2, H1N1, HAdV, and ZIKV) were mixed in fixed (n=5 per class per image, first dataset) and varying (n=2 to 8 per class per image, second dataset) proportions to emulate the situation where the four viruses were physically mixed on a slide. This operation was achieved using scripts written in MATLAB. For the second dataset, seven sets of images, each of 240 × 240 pixels, were generated. Each set contained 2 to 8 particles from each class, for a total of 1611 digitally mixed images. We used colocalized image pairs to train a deep convolutional neural network to map any phase image to a segmentation mask that provides a pixel-wise label for the background, SARS-CoV-2, H1N1, HAdV and ZIKV.

**Model Architecture and Training Strategies:** For the deep convolutional neural network, we picked a variant of U-Net ^16^ that has shown great performance on segmentation tasks with quantitative phase imaging (QPI) data in our previous works ^17-19^. The network contains an encoder path, a bottleneck connection and a decoder path (Figure 5a). The encoder path is responsible for extracting features from the input image via 4 stages of building blocks containing convolution layers interlaced by downsampling operations. Unlike the original U-Net, where the building block consists of convolutional operations only, we added in Batch Normalization layers ^20^ and residual connections ^21^ for faster convergence and better performance. We also reduced the number of filters in each layer of the network by a factor of 8. The numbers of convolutional filters in the 4 stages of the encoding path are set to 8, 16, 32, and 64 respectively. The convolutional filter sizes were set to 3 × 3 across the network except for the kernels used for residual connection, which were set to 1 × 1. Our model ended up having 0.8 million trainable parameters. Given an input digital-mixing image, our model will output a pixel-wise classification (Figure 5).

The model was trained with batches of images of size 160 × 160 pixels, randomly cropped from 240 × 240 images from our training set. The batch size was set to 20. The weights were optimized with the Adam optimizer against the categorical cross-entropy loss function:

$E=-\frac{1}{r\cdot c}\sum_{r=1}^{h} \sum_{c=1}^{w} \sum_{k=1}^{5} \delta(y[r][c]=k)\cdot\log(\mathop{y}\limits^{\wedge} [r][c][k])$ [7]

where $h$ and $w$ represent the number of rows and columns in the image. $\delta$ is the indicator function, which evaluates to 1 if $y[r][c]$, true label of the pixel $(r,c)$, is $k$. This loss function takes the average of the negative log-likelihood for the target class across every pixel in one image. It penalizes the model when it infers a small probability for the target class.

The model was implemented using Python and TensorFlow ^22^. The training was performed on an NVIDIA GTX 1070 GPU with 8 GB memory. For the first dataset, approximately 1200 virus particles were cropped out manually, using FIJI (ImageJ). We used over half of the selected virus particles to synthesize 32, 240 × 240 test images as the test dataset. Each of these images contained 20 virus particles equally distributed among SARS-CoV-2, H1N1, HAdV and ZIKV. For the training dataset, each virus particle was either rotated or flipped before placed onto the background image. We generated (with repetition of particles) 500 images for training and 50 for validation. The neural network we used was similar to the one explained previously (main text Figure 5), except that the number of trainable parameters is larger (Figure S12a) to account for the addition of background signal in the dataset.


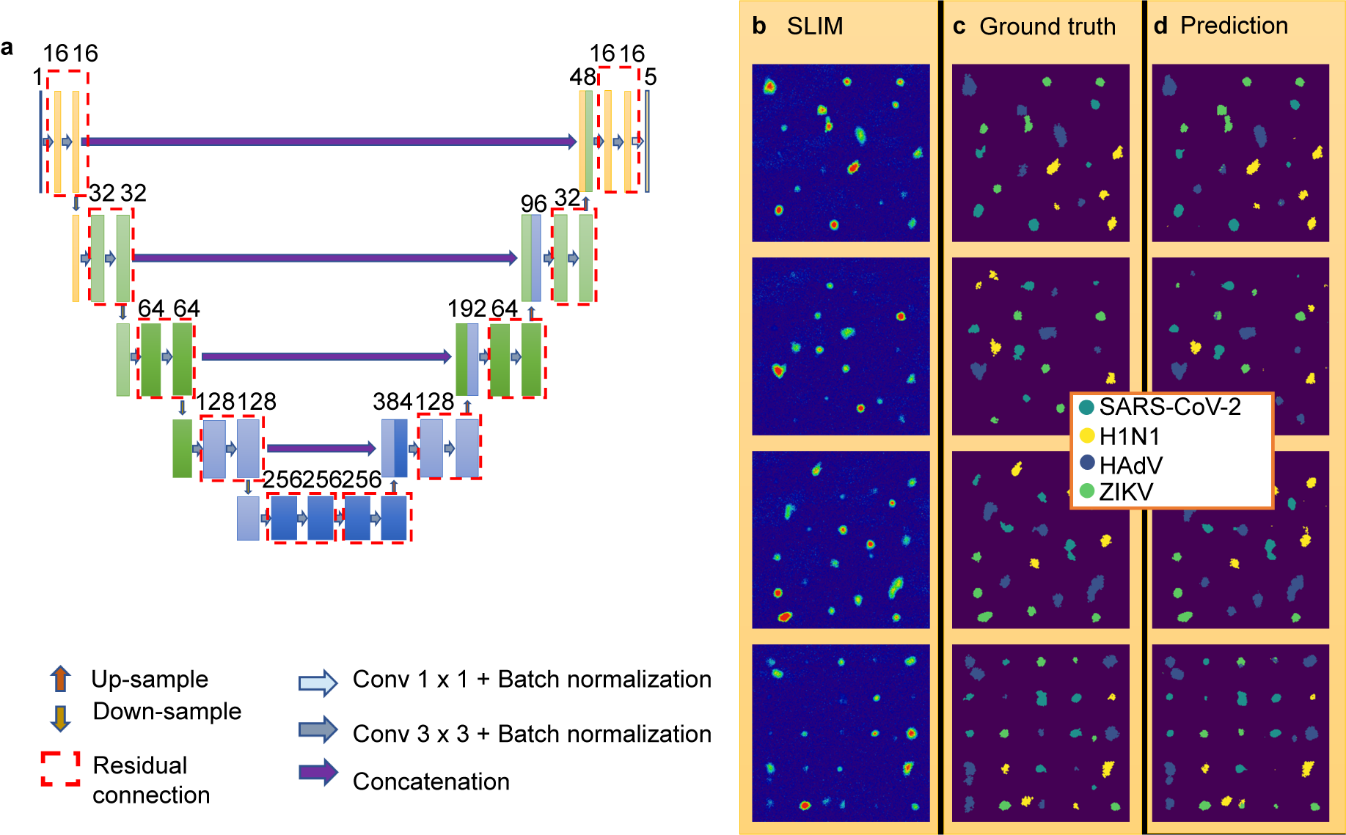


**Figure S12. Training a deep neural network to perform classification on the first digital-mixing dataset:** a. We used a modified version of U-Net for this semantic segmentation task. Besides reducing the number of parameters in the network to around 3 million, we also added in residual connection and batch normalization for faster convergence. Model inference on images from the validation set and the test set. b. Synthesized images of mixed virus particles. c. ground truth label. d. model inference.

Since pixel-wise segmentation accuracy did not clearly reflect the number of virus particles correctly and wrongly classified, we also introduced a particle-wise post-processing step ^23-25^. During the post-processing, we computed the average 5-class probability distribution across each virus particle:

$\mathop{p}\limits^{\wedge} (R)=\frac{1}{|R|}\sum_{r\in R} p(r)$ [8]

is a vector that denotes the model’s raw prediction for pixel $r$. Each entry in this vector gives the probability of pixel $r$ belonging to one of the 5 classes. Since our model used a softmax activation function as the output layer, the entries in $p(r)$ sums up to 1. $R$represents a set of pixels that belong to the same virus particle. We enforced, via this post-processing strategy, the instance-level information onto the model’s prediction such that the model will provide a unified label for one virus particle (Figure S13).

**Network Performance**

First dataset: SLIM images, ground truth and predictions are shown in Figures S12b-d respectively. After the application of post-processing (Figure S13), we plotted the one-versus-all ROC curve and confusion matrix (Figure S14). AUC values for four viruses are: 98% for SARS-CoV-2, 98% for H1N1, 96% for HAdV and 97% for ZIKV (Figure S14a). This model attained 0.80 precision and 0.88 recall for SARS-CoV-2, 0.82 precision and 0.73 recall for H1N1, 0.88 precision and 0.78 recall for HAdV, and 0.82 precision and 0.84 recall for ZIKV (Figure S14b).


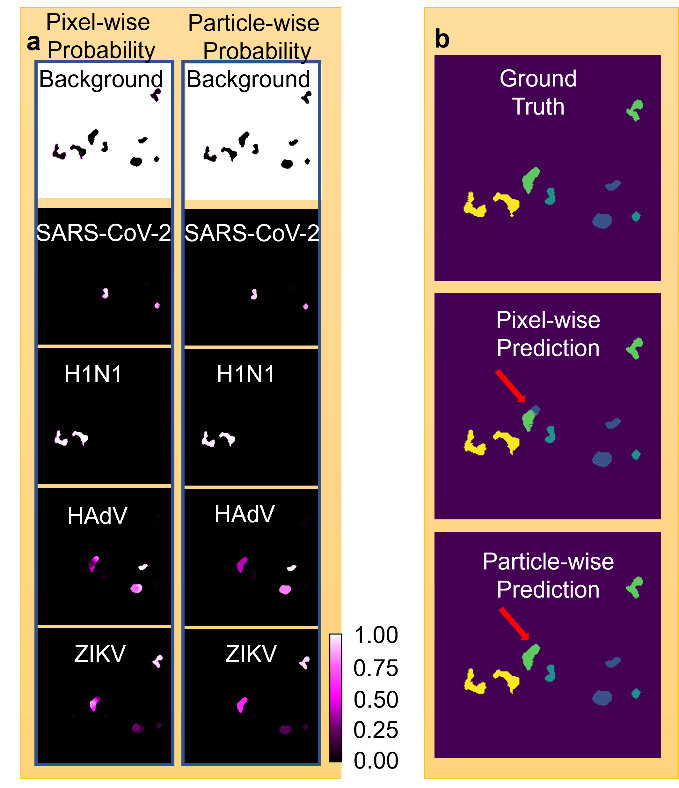


**Figure S13. Post-processing to enforce particle-level consistency**. a. To ensure all pixels in one virus particle has the same predicted label, we performed connected component analysis and averaged the probability distribution within each connected component. Left column: raw probability prediction; right column: probability distribution after post-processing. b. After post-processing, the predicted segmentation map no longer had different labels within one particle-region. This enabled us to compute, on an instance-level, the performance of our model.


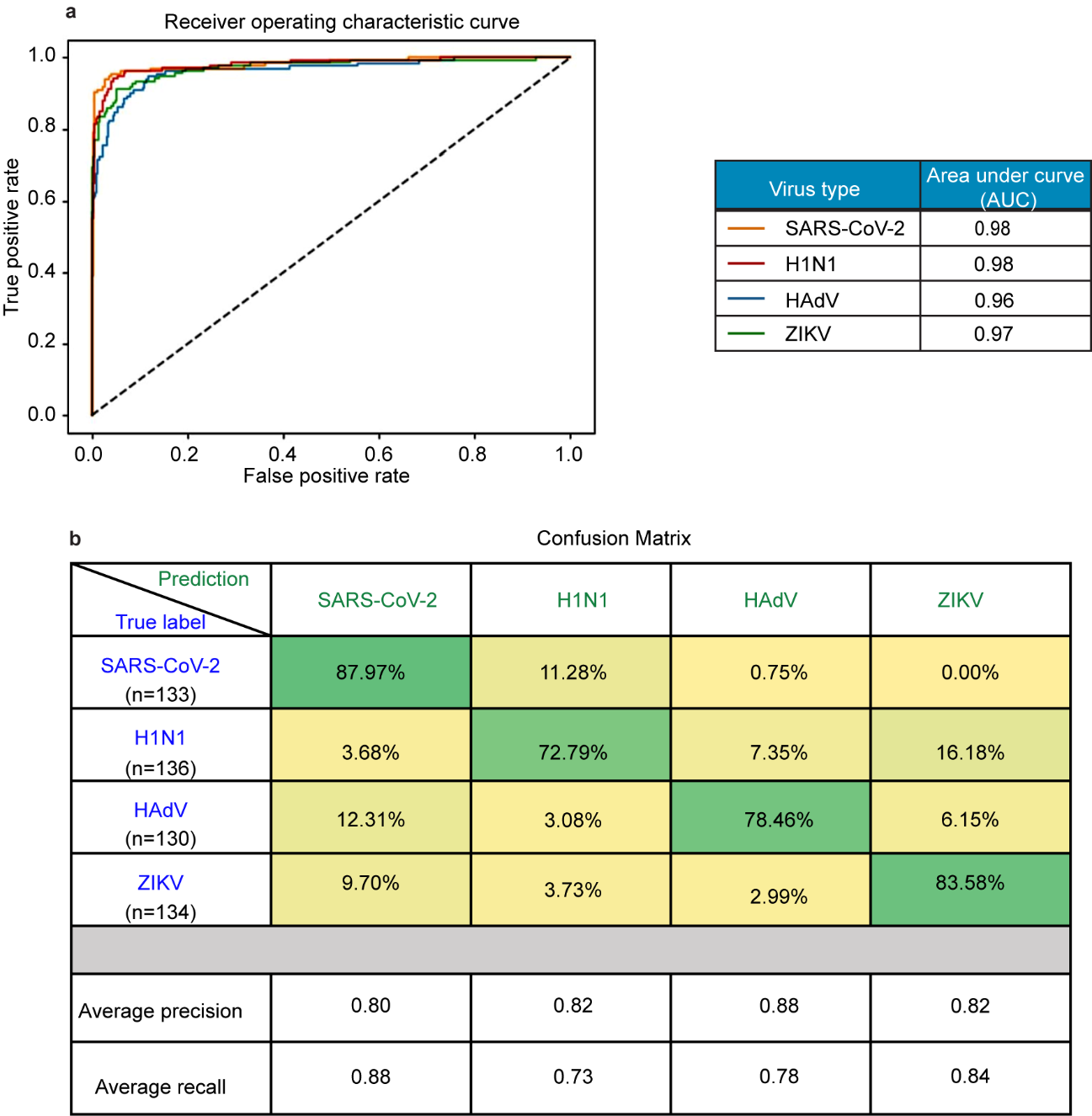


**Figure S14. Model performance on the first test dataset (consisting of 32 240 × 240 images)**. a. The receiver operating characteristic (ROC) curve of the model on the test dataset. The model achieved over 0.96 area-under-curve (AUC) for all 4 virus types on the test dataset. The area-under-curve (AUC) for each class is computed by setting that class as label 1 and all other class labels (background and the 3 remaining virus types) as label 0. b. The confusion matrix of the model inference on the test dataset. Each row represents the ground truth label while each column represents the prediction. For visualization purposes, each entry in the confusion matrix was normalized with respect to the number of true labels (sum of each row). The precision and recall are averaged across all images in the test dataset. Both the ROC curve and the confusion matrix are evaluated on a per-particle level.

Second dataset: The model was trained for 300 epochs. We set the initial learning rate to be 8e-5 and gave the model a warm up period ^26^ of 5 epochs. During the first 5 epochs, the learning rate increased linearly from 0 to 8e-5. After the warm up period, we implemented a simplified version of the cosine annealing strategy ^27,28^ and gradually turned down the learning rate to 0 for better convergence. This model had a categorical cross-entropy loss of 0.03 on the validation dataset after 300 epochs of training. The model weights that gave the smallest loss value on the validation dataset were selected as our end model and used for evaluation.

Our model was evaluated on a test dataset consisting of 564 unseen images. Each of these images contained 8 - 32 virus particles randomly placed on a 240 × 240 blank image.

The same post-processing step as used for the first dataset was used for this dataset too and was implemented using the connected component analysis tool from scikit-image ^29^. The model inference achieved on average 0.8 precision and 0.85 recall for SARS-CoV-2 particles in the test dataset after post-processing (Figure S15).


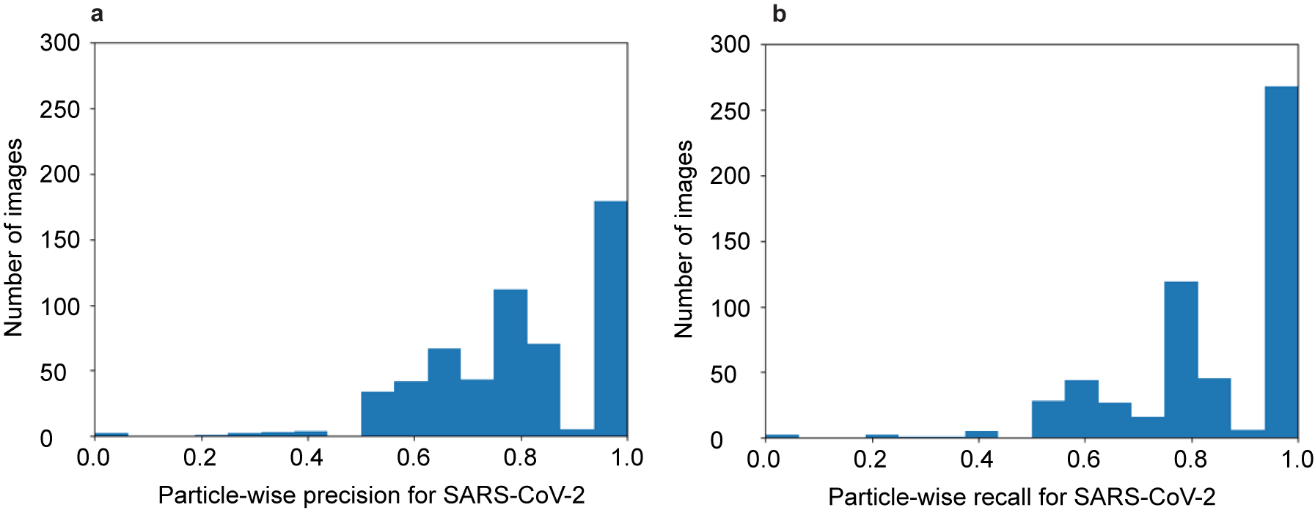


**Figure S15. Model Performance for SARS-CoV-2 with post-processing on the second dataset**. a. Histogram of particle-wise precision for SARS-CoV-2 evaluated on all 564 images in the test dataset. The average precision is 0.80. b. Histogram of particle-wise recall for SARS-CoV-2 evaluated on all 564 images in the test dataset. The average recall is 0.85.

All calculations are performed in Python using the scikit-learn library ^30^. Figure S16 shows the loss convergence for both our first (manual, Figure S16a) and second (automatic, Figure S16b) datasets.


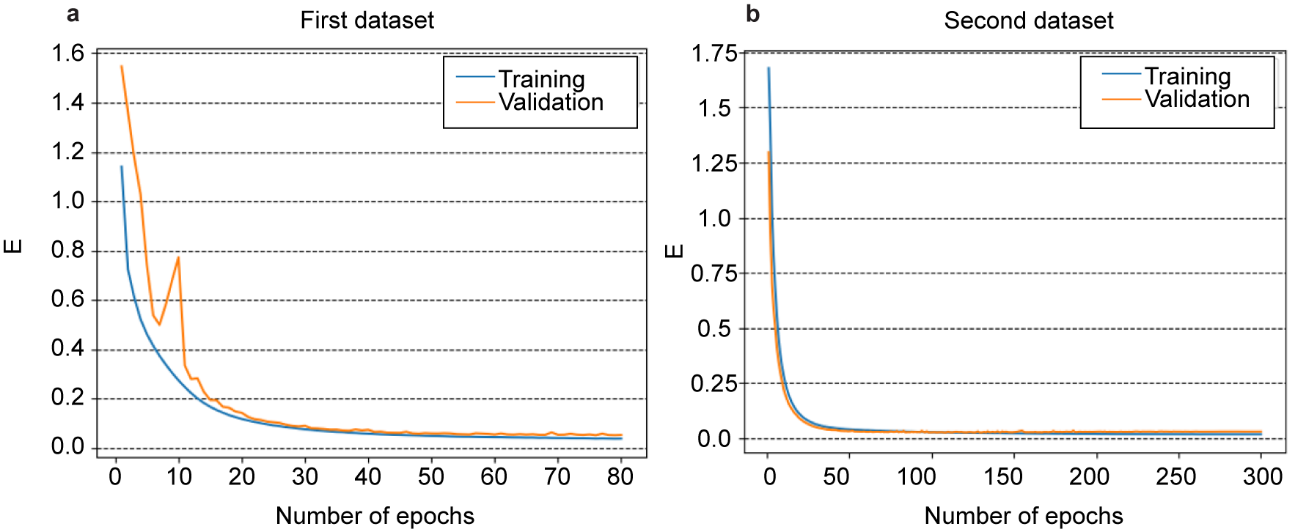


**Figure S16. Learning Curve Plot**. a. The learning curve plot of our model developed for the first dataset. b. The learning curve plot of our model developed for the second dataset. Both plots showed a good convergence between the validation loss and training loss of our models, indicating that our models did not underfit or overfit. E represents categorical cross-entropy Loss.

The interpretability of deep learning models remains an active field of research^31^. To better understand the decision-making process of our trained model, we adopted the gradient-weighted class activation mapping (Grad-CAM) approach^32^ on our trained U-Net based models^33^, aiming to visualize what regions of our SLIM images play an important role in the segmentation of different types of virus particles. We passed images from the test dataset into our network. For any activation map of interest, $A_{k}$with $k$channels, we first compute the neuron importance weights $\alpha_{k}^{c}$ to quantify how crucial this activation map is to the detection of virus particles of class $c$ as followed^32^:

$$\alpha_{k}^{c}=\frac{1}{Z}\sum_{i} \sum_{j} \frac{\partial y^{c}}{\partial A_{i,j}^{k}}$$

[9]

, where $y^{c}$ denotes the probability score map for a virus type; $(i,j)$ denote the pixel index; and $Z$ denotes the total number of pixels in the image.

We then used these weights to average all $k$ activation maps for each class $c$ as followed^32^:

$$L^{c}=ReLU\left(\sum_{k} \alpha_{k}^{c}A^{k}\right)$$

[10]

This heat-map is low-resolution, with the same traverse dimension as our bottleneck activation maps: 15 × 15. We up-sampled this heat-map with bi-linear interpolation and overlaid it with the input SLIM image as shown in Figure S17.

Figure S17a shows an example image from the test dataset. Figure S17b presents the Grad-CAM heat-map for all 4 virus types: HAdV, SARS-CoV-2, ZIKV, and H1N1, respectively. We noticed that the Grad-CAM heat-map for ZIKV showed that the network’s attention was more uniformly distributed to the entire input field compared with the other three heat-maps. This might help explain the relatively lower performance the network achieved on the ZIKV particles.


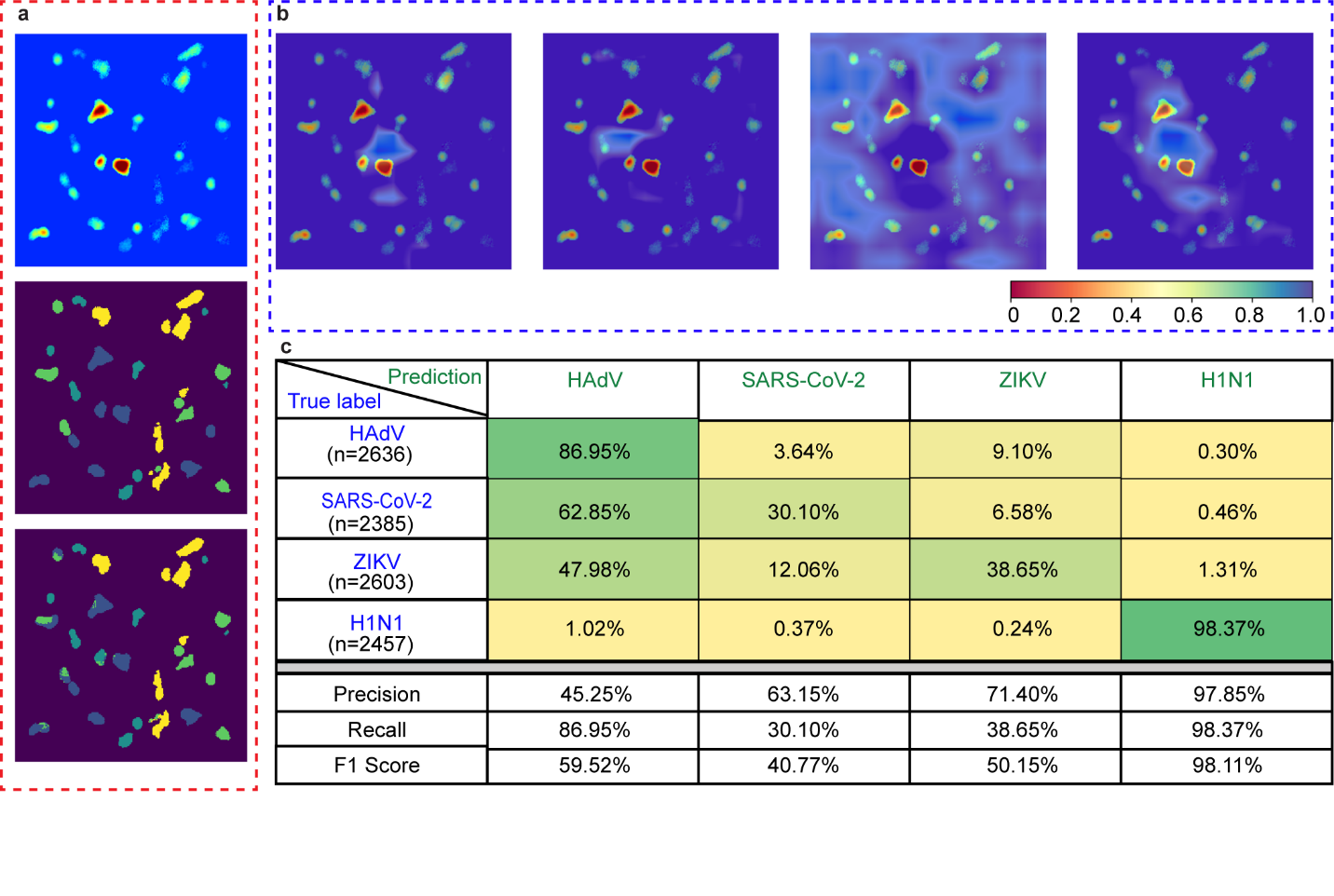


**Figure S17. Activation maps from the model** a. SLIM, ground truth and prediction images (top to bottom) b. Grad-CAM heatmaps for HAdV, SARS-CoV-2, ZIKV and H1N1 particles, respectively. **c.** Model performance after eroding the particle contours.

We also performed extra experiments to understand how the nano-morphological structures around the particles might affect the network inference. We curated another dataset by performing erosion on all virus particles (mask and SLIM image), which helped remove all the surface irregularities and textures along the particles in the SLIM image. Then we deployed the same trained model and evaluated its performance (Figure S17c). The network ended up with much more confusion among the HAdV, SARS-CoV-2, and ZIKV particles. Results indicate that more erosion is required for H1N1 to introduce significant drop in performance. However, since for erosion we used same structuring element for all four classes, further erosion would lead to disappearance of smaller particles from the dataset.

We also analyzed the predicted images, to verify that our model works on cluster of viruses as shown in Figure S18, where Figures S18a, b and c show SLIM, ground truth and predicted images respectively. Some of the obvious clusters are marked with red box in each case. Figure S18d shows the zoomed in images inside the red boxes in column a, with their deconvolved images in Figure S18e, showing the presence of multiple particles inside the clusters.


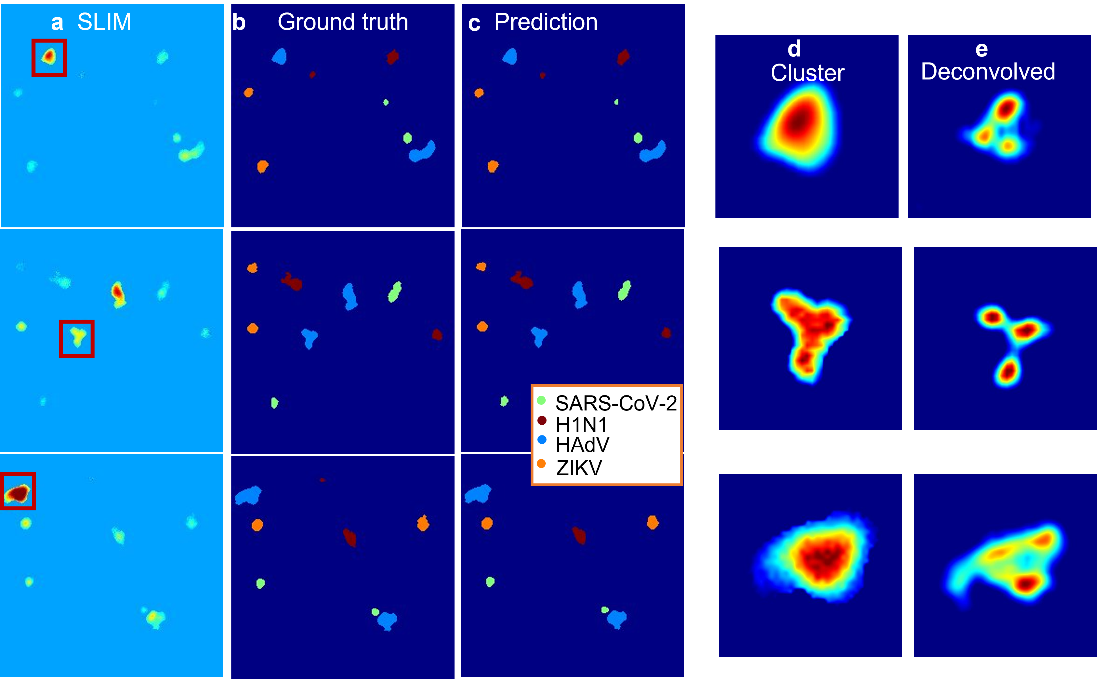


**Figure S18. Detection of virus clusters:** a SLIM image with some of the clusters marked with red boxes, b ground truth and c model prediction, d zoomed in images of clusters inside the red boxes in a, with their deconvolved images shown in e indicating the presence of multiple particles.

To test our technique in the presence of other sub-diffraction particles such as dust, we retrained a model (Efficientnetb0+Unet) on a dataset consisting of digitally mixed images of SARS-CoV-2, H1N1, HAdV, ZIKV and 25 nm polystyrene beads (representing sub-diffraction dust particles). Model performance is shown in Figure S19 showing that our model is able to detect and classify viruses and differentiate them from dust particles as well.


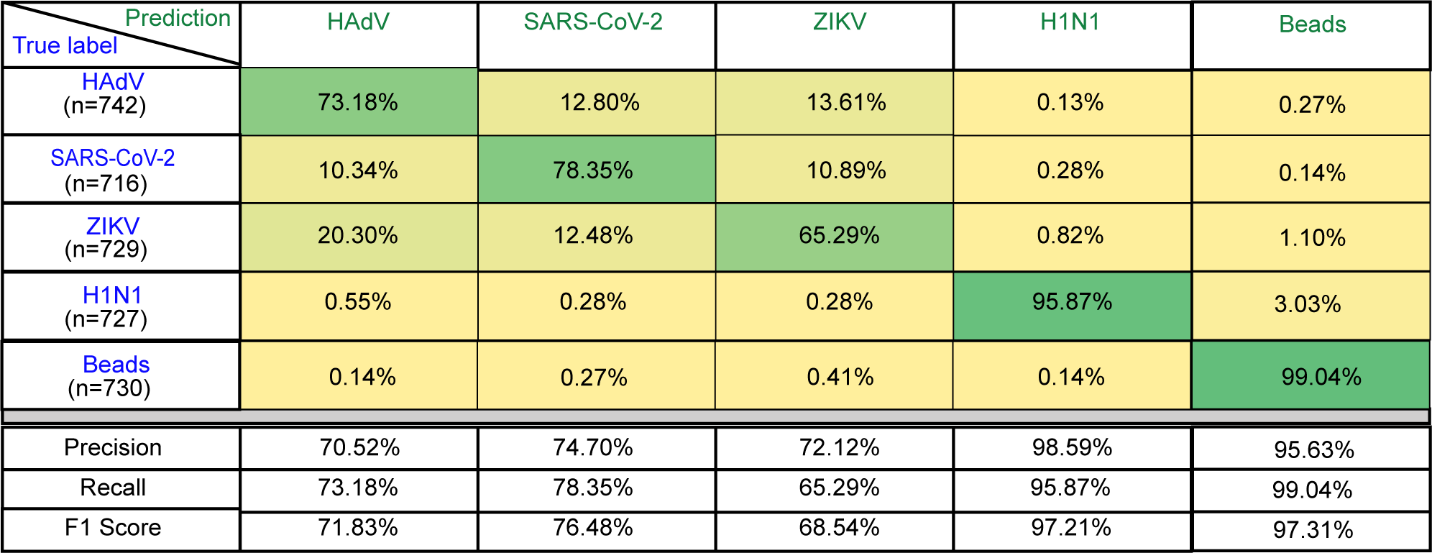


**Figure S19. Particle-wise Confusion Matrix:** on 240 x 240 test images for 5 particles, SARS-CoV-2, H1N1, HAdV, ZIKV and sub-diffraction polystyrene beads emulating dust and background particles.

As a part of the clinical translation, breath test utilizing our technique is proposed in Figure S20. A patient would breathe on a glass slide for 1-5 minutes. The glass slide will then be taken for SLIM imaging and inference. Random field of views will be chosen across the marked region of the slide (1 cm x 1 cm) for imaging. The whole imaging, inference and results will take about a minute.


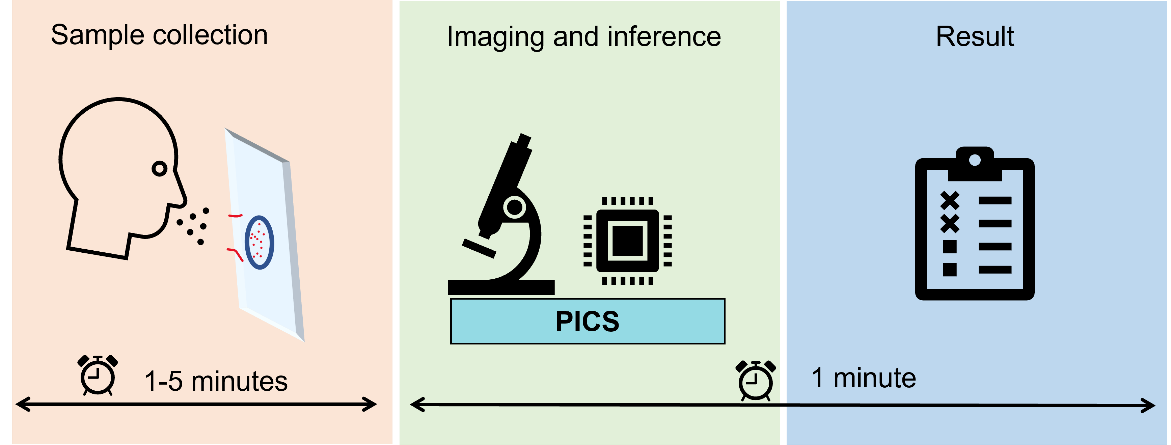


**Figure S20. Breath test schematic:** A patient would breathe on a glass slide, which can take at most 5 minutes and the subsequent imaging and inference will take about a minute.

To test the limit of detection of our method, we created digitally mixed images of 1 particle per class per image. The image size was now 2048 x 2048. Our model was able to detect single particle per class in the biggest field of view. The performance is shown in Figure S21.


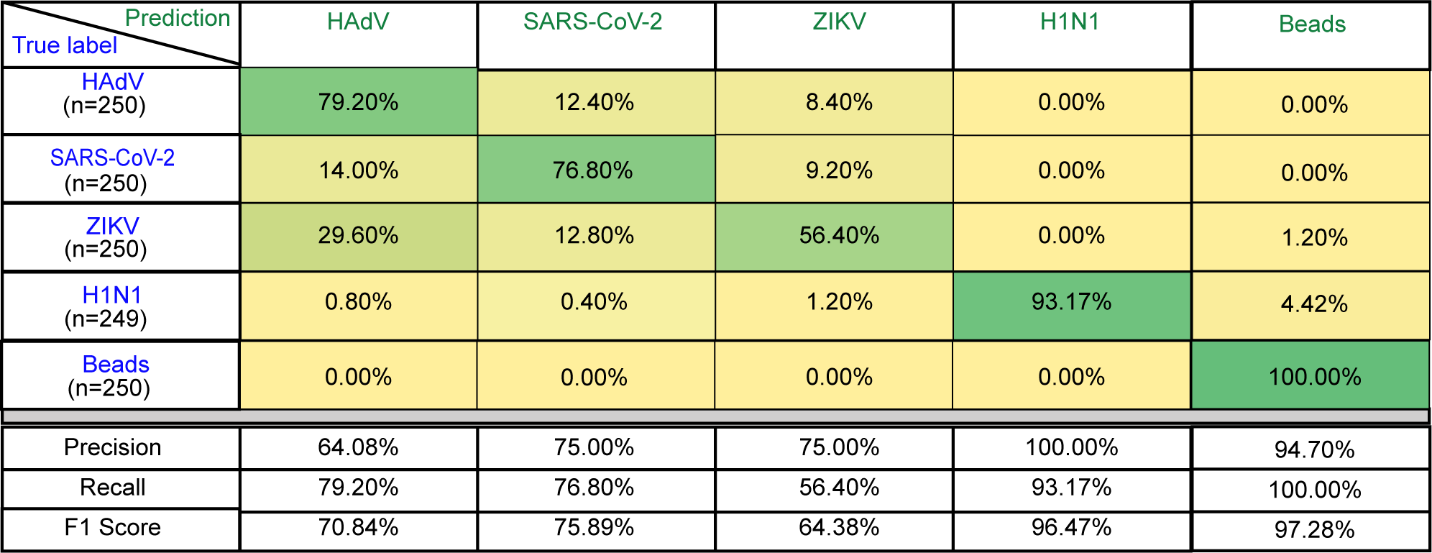


**Figure S21. Limit of detection:** Performance of model in case of 1 particle per class in a 2048 x 2048 image.

**Movie S1 (separate file).** Volumetric reconstruction of a group of SARS-CoV-2 particles with maximum projection emphasizing the irregular boundaries of the virus particles.

Movie S2 (separate file). Volumetric reconstruction of a group of H1N1 particles with maximum projection emphasizing the irregular boundaries of the virus particles and pleomorphic shapes of H1N1 particles.

Movie S3 (separate file). Volumetric reconstruction of a group of HAdV particles with maximum projection emphasizing the hexagonal shape of the virus particles.

Movie S4 (separate file). Volumetric reconstruction of a group of ZIKV particles with maximum projection emphasizing the relatively smoother surface of the virus particles.

Movie S5 (separate file). Volumetric reconstruction of two SARS-CoV-2 particles with z-evolution emphasizing the variation of visibility of ultrastructure with z position.

**SI References**

1 Cascella, M., Rajnik, M., Cuomo, A., Dulebohn, S. C. & Di Napoli, R. in *Statpearls [internet]* (StatPearls Publishing, 2020).

2 Ke, Z. *et al.* Structures and distributions of SARS-CoV-2 spike proteins on intact virions. *Nature*, 1-7 (2020).

3 Vazquez-Bravo, B., Gonçalves, K., Shisler, J. L. & Mariñas, B. J. Adenovirus replication cycle disruption from exposure to polychromatic ultraviolet irradiation. *Environmental science & technology* **52**, 3652-3659 (2018).

4 San Martín, C. Transmission electron microscopy and the molecular structure of icosahedral viruses. *Archives of biochemistry and biophysics* **581**, 59-67 (2015).

5 Sirohi, D. & Kuhn, R. J. Zika virus structure, maturation, and receptors. *The Journal of infectious diseases* **216**, S935-S944 (2017).

6 Pickett, B. E. e. a. *Zika Virus, in NIAID Virus Pathogen Database and Analysis Resource (ViPR)* 2012).

7 Caldas, L. A., Azevedo, R. C., da Silva, J. L. & de Souza, W. Microscopy analysis of Zika virus morphogenesis in mammalian cells. *Scientific Reports* **10**, 1-11 (2020).

8 Sirohi, D. *et al.* The 3.8 Å resolution cryo-EM structure of Zika virus. *Science* **352**, 467-470 (2016).

9 Sabbaghi, A., Miri, S. M., Keshavarz, M., Zargar, M. & Ghaemi, A. Inactivation methods for whole influenza vaccine production. *Reviews in medical virology* **29**, e2074 (2019).

10 Kojima, M. *et al.* Irradiation by a Combination of Different Peak-Wavelength Ultraviolet-Light Emitting Diodes Enhances the Inactivation of Influenza A Viruses. *Microorganisms* **8**, 1014 (2020).

11 McDevitt, J. J., Rudnick, S. N. & Radonovich, L. J. Aerosol susceptibility of influenza virus to UV-C light. *Applied and environmental microbiology* **78**, 1666-1669 (2012).

12 Harris, A. K. *et al.* Structure and accessibility of HA trimers on intact 2009 H1N1 pandemic influenza virus to stem region-specific neutralizing antibodies. *Proceedings of the National Academy of Sciences* **110**, 4592-4597 (2013).

13 Dey, N. *et al.* Richardson–Lucy algorithm with total variation regularization for 3D confocal microscope deconvolution. *Microscopy research and technique* **69**, 260-266 (2006).

14 Sage, D. *et al.* DeconvolutionLab2: An open-source software for deconvolution microscopy. *Methods* **115**, 28-41 (2017).

15 Popescu, G. *Quantitative phase imaging of cells and tissues*. (McGraw Hill Professional, 2011).

16 Ronneberger, O., Fischer, P. & Brox, T. in *International Conference on Medical image computing and computer-assisted intervention.* 234-241 (Springer).

17 Kandel, M. E. *et al.* Reproductive outcomes predicted by phase imaging with computational specificity of spermatozoon ultrastructure. *Proceedings of the National Academy of Sciences* **117**, 18302-18309, doi:10.1073/pnas.2001754117 (2020).

18 Kandel, M. E. *et al.* Multiscale assay of unlabeled neurite dynamics using phase imaging with computational specificity (PICS). *arXiv preprint arXiv:2008.00626* (2020).

19 Kandel, M. E. *et al.* PICS: Phase Imaging with Computational Specificity. *arXiv preprint arXiv:2002.08361* (2020).

20 Ioffe, S. & Szegedy, C. Batch normalization: Accelerating deep network training by reducing internal covariate shift. *arXiv preprint arXiv:1502.03167* (2015).

21 He, K., Zhang, X., Ren, S. & Sun, J. in *Proceedings of the IEEE conference on computer vision and pattern recognition.* 770-778.

22 Abadi, M. *et al.* Tensorflow: Large-scale machine learning on heterogeneous distributed systems. *arXiv preprint arXiv:1603.04467* (2016).

23 Rehn, E., Rehn, A. & Possemiers, A. Fossil charcoal particle identification and classification by two convolutional neural networks. *Quaternary Sci Rev* **226**, 106038 (2019).

24 Caicedo, J. C. *et al.* Evaluation of deep learning strategies for nucleus segmentation in fluorescence images. *Cytometry Part A* **95**, 952-965 (2019).

25 Hu, C. *et al.* Label-free cell viability assay using phase imaging with computational specificity. *bioRxiv* (2020).

26 Goyal, P. *et al.* Accurate, large minibatch sgd: Training imagenet in 1 hour. *arXiv preprint arXiv:1706.02677* (2017).

27 Loshchilov, I. & Hutter, F. Sgdr: Stochastic gradient descent with warm restarts. *arXiv preprint arXiv:1608.03983* (2016).

28 He, T. *et al.* in *Proceedings of the IEEE Conference on Computer Vision and Pattern Recognition.* 558-567 (2019).

29 Van der Walt, S. *et al.* scikit-image: image processing in Python. *PeerJ* **2**, e453, doi:10.7717/peerj.453 (2014).

30 Pedregosa, F. *et al.* Scikit-learn: Machine learning in Python. *J Mach Learn Res* **12**, 2825-2830 (2011).

31 Lipton, Z. C. The Mythos of Model Interpretability: In machine learning, the concept of interpretability is both important and slippery. *Queue* **16**, 31-57 (2018).

32 Selvaraju, R. R. *et al.* in *Proceedings of the IEEE international conference on computer vision.* 618-626 (2017).

33 Vinogradova, K., Dibrov, A. & Myers, G. Towards interpretable semantic segmentation via gradient-weighted class activation mapping. *arXiv preprint arXiv:2002.11434* (2020).
